# Rab10-associated tubulation as an early marker for biogenesis of the assembly compartment in cytomegalovirus-infected cells

**DOI:** 10.3389/fcell.2024.1517236

**Published:** 2025-01-10

**Authors:** Hana Mahmutefendić Lučin, Igor Štimac, Marina Marcelić, Matej Skočaj, Berislav Lisnić, Alen Omerović, Ivona Viduka, Barbara Radić, Ljerka Karleuša, Gordana Blagojević Zagorac, Martina Deželjin, Antonija Jurak Begonja, Pero Lučin

**Affiliations:** ^1^ Department of Physiology, Immunology and Pathophysiology, Faculty of Medicine, University of Rijeka, Rijeka, Croatia; ^2^ University North-University Center Varaždin, Varaždin, Croatia; ^3^ Department of Biology, Biotechnical faculty, University of Ljubljana, Ljubljana, Slovenia; ^4^ Center for Proteomics, Faculty of Medicine, University of Rijeka, Rijeka, Croatia; ^5^ Institute of Virology, Hannover Medical School, Hannover, Germany; ^6^ Division of Molecular Biology, Ruđer Bošković Institute, Zagreb, Croatia; ^7^ Faculty of Biotechnology and Drug Development, University of Rijeka, Rijeka, Croatia

**Keywords:** cytomegalovirus, assembly compartment, beta-herpesvirus secondary envelopment, RAB10, tubular recycling endosomes

## Abstract

**Introduction:**

Cytomegalovirus (CMV) infection reorganizes early endosomes (EE), recycling endosome (RE), and trans-Golgi network (TGN) and expands their intermediates into a large perinuclear structure that forms the inner part of the cytoplasmic assembly complex (AC). The reorganization begins and results with the basic configuration (known as pre-AC) in the early (E) phase of infection, but the sequence of developmental steps is not yet well understood. One of the first signs of the establishment of the inner pre-AC, which can be observed by immunofluorescence, is the accumulation of Rab10. This study aims to investigate whether Rab10-positive domain (Rab10-PD) is expanded during the E phase of infection.

**Methods:**

We performed long-term live imaging of EGFP-Rab10 with epifluorescence imaging-enhanced digital holotomographic microscopy (DHTM), confocal imaging of known Rab10 interactors and identification of important Rab10 interactors with the proximity-dependent biotin identification assay (BioID). The accumulation of Rab10-PD was analyzed after knock-down of EHBP1 and Rabin8, two proteins that facilitate Rab10 recruitment to membranes, and after blocking of PI(4,5)P2 by PI(4,5)P2-binding protein domains.

**Results:**

Our study shows the gradual expansion of Rab10-PD in the inner pre-AC, the association of Rab10 with EHBP1 and MICAL-L1, and the dependence of Rab10-PD expansion on EHBP1 and PI(4,5)P2 but not Rabin8, indicating the expansion of EE-derived tubular recycling endosome-like membranes in the pre-AC. Silencing of Rab10 and EHBP1 suggests that Rab10-PD expansion is not required for the establishment of the inner pre-AC nor for the expansion of downstream tubular domains.

**Conclusion:**

The present work characterizes one of the earliest sequences in the establishment of pre-AC and suggests that subsets of EE-derived tubular membranes may serve as the earliest biomarkers in pre-AC biogenesis. Our study also indicates that the pre-AC biogenesis is complex and likely involves multiple parallel processes, of which Rab10-PD expansion is one. Our experiments, particularly our silencing experiments, show that Rab10 and EHBP-1 do not play a significant role in the later stages of inner pre-AC biogenesis or in the expansion of downstream tubular domains. A more comprehensive understanding of the tubular domain expansion remains to be established.

## 1 Introduction

Beta-herpesviruses (BHVs) infect a large proportion of the human population and are associated with a variety of pathophysiologic conditions (Crawford, 2023). Among BHVs, cytomegalovirus (CMV), also known as human herpesvirus 5 (HHV5 or HCMV), has received the most attention, and most knowledge about BHV has been gained through CMV research. Several approaches are currently being developed to combat HCMV infections, including an approach of targeting host cell proteins known as host-directed therapy (Kumar et al., 2020).

CMV infection completely reorganizes the infected cells and leads to many complex cytopathic effects, including the formation of two megastructures, the cytoplasmic assembly complex (AC) and the nuclear replication centers (Wofford et al., 2020; Sanchez and Britt, 2022; Turner and Mathias, 2022). The AC represents a part of the infected cell reorganized membrane system (ICRMS), which includes the relocation of the Golgi into a ring-like configuration that encloses a large perinuclear region containing early endosomes (EE), recycling endosomes (RE), the trans-Golgi network (TGN), and expanded membrane intermediates of the EE-RE/ERC-TGN interface (Das et al., 2007; Das and Pellett, 2011; Lučin et al., 2020). This structure, which is as large as the nucleus of the infected cell, is likely the site where the final steps of CMV virion assembly take place (Wofford et al., 2020), and it appears that the entire ICRMS contributes to virion egress (Wofford et al., 2020; Lučin et al., 2023). The majority of ER and LE membranes are extruded from the AC to the cell periphery (Beltran et al., 2016; Lučin et al., 2020).

Most studies on ICRMS have focused on the AC when this structure is fully established and the entire CMV gene repertoire is expressed, i.e. 96-120 h post-infection (hpi) in HCMV-infected cells (Das et al., 2007; Das and Pellett, 2011; Wofford et al., 2020). Reorganization is initiated early in infection and the basic configuration of the AC is established in the early (E) phase before viral DNA replication and late (L) gene expression (Sanchez et al., 2000; Das et al., 2007; Beltran et al., 2016; Rebmann GM et al., 2016; Procter et al., 2018; Taisne et al., 2019). In HCMV-infected cells, the establishment of the basic configuration takes 48 h or longer (Beltran et al., 2016; Wofford et al., 2020). However, the sequence of AC establishment has not yet been studied in detail and the developmental steps are poorly understood. Similar organizational changes occur in fibroblast-like cells infected with murine CMV (MCMV). In MCMV-infected cells, the AC is formed much earlier: the basic configuration is rapidly established between 5 and 7 hpi and evolves during the E phase of infection into a structure termed pre-AC (Taisne et al., 2019; Lučin et al., 2020). This is followed by the expression of numerous viral L genes that load pre-AC to establish AC, which matures further in the replication cycle, after viral DNA replication begins at 15–16 hpi (Lučin et al., 2020).

The membrane system reorganization is driven by viral gene products that target membrane-shaping regulatory host cell factors and thereby directly modulate their function (Das et al., 2014; Hook et al., 2014; Bughio et al., 2015; Dietz et al., 2018; Wu et al., 2020). This can also be achieved indirectly by stimulating their degradation (Lin et al., 2021) or by triggering signaling events that regulate membrane traffic (Cruz and Buchkovich, 2017). The early establishment of the basic configuration suggests an essential function of CMV E genes in the process, which actively remodel the membrane domains of the endosomal and secretory membrane system and reorganize membrane flux (Krzyzaniak et al., 2009; Tomaš et al., 2010; Hook et al., 2014; Zeltzer et al., 2018). Studies on HCMV-infected cells showed that several HCMV gene products, including pUL48 and pUL103 (Das et al., 2014), pUL97 (Azzeh et al., 2006), and gpUL132 (Wu et al., 2020), contribute to the formation of the ring-shaped configuration. Studies focusing on individual host cell factors that regulate the organization of membrane flux (Crump et al., 2003; Krzyzaniak et al., 2009; Tandon et al., 2009; Fraile-Ramos et al., 2010; Cepeda and Fraile-Ramos, 2011; Indran and Britt, 2011; Gudleski-O’Regan et al., 2012; Sharon-Friling and Shenk, 2014; Pavelin et al., 2017; Maschkowitz et al., 2018; Procter et al., 2018; Taisne et al., 2019; Hashimoto et al., 2020; Yang et al., 2022) and broader screening (McCormick et al., 2018; Turner et al., 2020) suggest that the virus targets diverse host cell machinery. The transcriptome studies showed that both HCMV (Hertel and Mocarski, 2004; Close et al., 2018) and MCMV (Juranic Lisnic et al., 2013; Lučin et al., 2020) affect the transcriptional activity of a large number of host cell genes related to membrane flux. In HCMV-infected cells (Weekes et al., 2014; Beltran et al., 2016; Jean Beltran et al., 2017a), many host cell proteins associated with membrane traffic are upregulated or downregulated, leading to an imbalance that may be associated with such extensive reorganization. This imbalance leads to an organization of membrane domains that differs from that observed in uninfected cells (Beltran et al., 2016; Tyl et al., 2022), and even to a distinct organelle structure (Jean Beltran et al., 2017b; 2017a; Zeltzer et al., 2018), which is associated with a displacement of many host cell proteins from the conventional organelles established in uninfected cells (Cook and Cristea, 2019). Therefore, it is becoming increasingly difficult to transfer the classical configuration of the membrane system established in uninfected cells to ICRMS. Accordingly, the physiology of membrane traffic is also likely to be altered and difficult to compare with that in uninfected cells. It is therefore essential to investigate the transport pathways in ICRMS in more detail and to compare them with the constantly growing knowledge about the membrane system of non-infected cells.

Studies on the part of the ICRMS known as the AC usually divide this structure into an outer AC (and the outer pre-AC in the E phase of infection) consisting of tangled and expanded Golgi elements surrounding the inner AC, which consists of expanded EE-, RE/ERC- and TGN-derived membrane structures in a large perinuclear area (Das and Pellett, 2011; Lučin et al., 2020; Wofford et al., 2020). The membrane structures within the inner AC are used for the final assembly of the CMV virions, the so-called secondary envelopment, and for the construction of an intracellular pathway for their exit from the cell (Wofford et al., 2020). Our studies on MCMV-infected cells have shown that unlinking and displacement of the Golgi and perinuclear expansion of the EE and REs/ERC domains are among the first cellular events that can be identified by immunofluorescence as signs of ICRMS and subsequent development of the AC (Lučin et al., 2020; Štimac et al., 2021). In the inner pre-AC, we first observed pericentriolar accumulation of membranes decorated with the small GTPase Rab10 (Karleuša et al., 2018; Lučin et al., 2020), and used it as an indicator of the earliest events of pre-AC biogenesis (Marcelić et al., 2021; Pavišić et al., 2021; Štimac et al., 2021; Štimac et al., 2024). However, little biochemical evidence has been provided for its enhanced membrane mobilization.

Rab10 is a versatile Rab that can be activated at different cellular localizations and can be involved in various processes at different developmental stages of the membrane system (Chua and Tang, 2018). Although Rab10 can be activated at the ER (Babbey et al., 2006; English and Voeltz, 2013; Liu et al., 2013), the ER is excluded from the inner pre-AC (Sanchez et al., 2000; Das and Pellett, 2011; Lučin et al., 2020), and the pericentriolar accumulation of Rab10 indicates the expansion of tubular intermediates at EEs or within the ERC (Karleuša et al., 2018; Lučin et al., 2020; Štimac et al., 2021). Rab10 can be activated on Rab5-positive EEs, where Rab5 and Rab10 can form a regulatory cascade in which Rab5 recruits the GDP-GTP exchange factor (GEF) to activate Rab10 (Liu et al., 2018), and Rab10 recruits the GTP hydrolysis activating protein (GAP) that inactivate Rab5 (Liu and Grant, 2015). Rab10 can also be activated simultaneously with Rab11 at EE as they can share the GEF (Xiong et al., 2012), which promotes the development of domains associated with the sorting of different endocytic cargo. Activation of Rab10, in cooperation with Rab22a, drives the development of tubular recycling endosomes (TREs) (Etoh and Fukuda, 2019; Farmer et al., 2021), which sort and recycle clathrin-independent endocytic cargo (CIE) to the PM (Xie et al., 2016). In cells forming TREs, Rab10 is recruited together with EHBP1 to phosphatidylinositol (4,5)bisphosphate (PI(4,5)P2)-enriched membranes (Farmer et al., 2021). The recruitment of Rab10 to TREs is facilitated by the recruitment of the Rab10 effector EHBP1 (Farmer et al., 2021). It appears that in most cells, the established Rab10 domain at the EEs is highly dynamic, the generated tubules are rapidly converted into transport intermediates and Rab10 is rapidly removed from the membranes and released into the cytosol (Babbey et al., 2006). Rab10 function may also be associated with the downstream population of tubular membranes that process and recycle CIE cargo known as Arf6/Rab8-REs (Kobayashi and Fukuda, 2013). However, Rab10 can also be activated downstream in the recycling tract, at a subset of Rab11-REs, by Rab11, which recruits Rabin8, GEF for Rab8 and Rab10 (Homma and Fukuda, 2016).

The Rab10-positive domain (Rab10-PD) in the inner pre-AC of MCMV-infected cells may be co-opted by the infection for the needs of the CMV replication cycle and could be a biomarker for pre-AC biogenesis. Therefore, it would be important to determine whether Rab10 is over-recruited to membranes and Rab10-PD expanded within the inner pre-AC. To do so, we performed long-term live imaging of GFP-Rab10 using digital holotomographic microscopy (DHTM) in combination with epifluorescence, confocal imaging of known Rab10 interactors and identification of important Rab10 interactors using the BioID (proximity-dependent biotin identification) assay (Roux et al., 2012). We also analyzed perinuclear Rab10 accumulation after knock-down of EHBP1 and Rabin8, two proteins required for Rab10 recruitment to membranes (Farmer et al., 2021), and after blocking PI(4,5)P2-rich membrane domains. Our study demonstrates the gradual expansion of Rab10-PD in the inner pre-AC as Rab10 associates with EHBP1 and MICAL-L1. The formation of the expanded Rab10-PD requires EHBP1 and PI(4,5)P2, but not Rabin8. These data suggest the expansion of a subset of EE-derived TRE-like membranes in pre-AC, which may serve as the earliest biomarker of pre-AC biogenesis.

## 2 Materials and methods

### 2.1 Cell lines and cell culture

The NIH 3T3 (ATCC CRL-163) and Balb 3T3 (clone A31, ATCC CCL-163) murine fibroblast-like cell lines were propagated in 10 cm Petri dishes and divided into appropriate plates once they were 80%–90% confluent. Cells were cultured in Dulbecco’s Modified Eagle’s Medium (DMEM) supplemented with 10% fetal bovine serum (FBS), 2 mM L-glutamine, 100 mg/mL streptomycin and 100 U/mL penicillin (all reagents from Gibco/Invitrogen, Grand Island, NY, United States) at 37°C and 5% CO_2_.

### 2.2 Murine cytomegalovirus and infection procedures

For infection, we used Δm138-MCMV (ΔMC95.15), a recombinant virus with the deletion of the viral fcr1 (m138) gene (Crnković-Mertens et al., 1998). In some experiments, we used the MCMV wild-type strain Smith (ATCC VR-194; American Type Culture Collection [ATCC]). The production of MCMV stocks and infection conditions were according to the standard procedures as described before (Brizić et al., 2018). In brief, adherent cells were infected with 1 PFU/cell, and a multiplicity of infection (MOI) of 10 was achieved by centrifugal enhancement (2000 rpm/15 min). Immediate-Early-1 protein (pIE1) monitoring by immunofluorescence or Western blot was performed regularly to verify infection as previously described (Lučin et al., 2020).

### 2.3 Antibodies and reagents

The following monoclonal (mAb) or polyclonal (pAb) antibodies against host cell proteins were used in this study: rabbit mAb (IgG) to Rab10 (Cat.No. 8127; Cell Signaling Inc, Danvers, MA, United States), rabbit pAb (IgG) to Rab3IP/Rabin8 (Cat.No. 12321-1-AP; Proteintech, Manchester, United Kingdom), rabbit mAb (IgG) to Rab5 (Cat.No. 3547; Cell Signaling Inc, Danvers, MA, United States), rabbit pAb (IgG) to TBC1D4/AS160 (Cat.No. LS-C368534; LS-Bio, Shirley, MA, United States), rabbit pAb (IgG) to TBC1D2 (Cat.No. LS-C157145; LS-Bio, Shirley, MA, United States), rabbit pAb (IgG) to EHBP1 (Cat.No. 17637-1-AP; Proteintech, Manchester, United Kingdom), rabbit pAb (IgG) to ACAP1/CENTB1 (Cat.No. orb182538; Biorbyt, Cambridge, United Kingdom), mouse mAb (IgG) to ACAP2/CENTB2, (Cat. No. Sc-376150; Santa Cruz Biotechnology, Dallas, United States), rabbit pAb (IgG) to MICALL1 (Cat.No. orb537847; Biorbyt, Cambridge, United Kingdom), mouse mAb (IgG1) to GM130 (Cat. No. 610823, BD Biosciences, Franklin Lakes, NJ, United States), and mouse mAbs to β-actin (IgG_1_; Cat. No. MAB150; Millipore, Burlington, Massachusetts, United States) and to HA (Cat.No. 51064-2-AP; Proteintech, Manchester, United Kingdom). W6/32 mAb (mouse IgG2a, ATCC HB-95) that react with human but not with mouse MHC class I proteins was used as hybridoma supernatant. Monoclonal antibodies against MCMV protein, I.E.,1 (pm123/pIE1) included clones CROMA101 (IgG_1_) and, I.E.,1.01 (IgG_2a_), produced by the University of Rijeka Center for Proteomics, (https://products.capri.com.hr/shop/?swoof=1&pa_reactivity=murine-cytomegalovirus; accessed on 25 October 2024).

For immunofluorescence analysis, we used Alexa Fluor (AF)-conjugated (AF^488^, AF^594^, and AF^555^) secondary Abs to mouse IgG_2a_, mouse IgG_1_, and rabbit IgG (Molecular Probes, Leiden, Netherlands), and AF^680^-conjugated secondary Abs to IgG_1_ and IgG_2a_ (Jackson Laboratory, Bar Harbor, ME, United States). Streptavidin-AF^488^ was from Thermo Fisher Scientific (Waltham, MA, United States). For Western blot analysis, we used HRP-conjugated goat anti-rabbit and goat anti-mouse Abs (Jackson Laboratories, Bar Harbor, ME, United States). DAPI (4,6-diamidino-2-phenylindole dihydrochloride) was obtained from Thermo Fisher Scientific (Waltham, MA, United States; Cat. No. D1306), puromycin from Santa Cruz Biotechology Inc (Dallas, United States), and propidium iodide and biotin from Sigma-Aldrich Chemie GmbH (Schnelldorf, Germany).

### 2.4 Immunofluorescence and confocal microscopy

The 60%–70% confluent cells grown on coverslips in 24-well plates were MCMV infected, fixed in 4% paraformaldehyde (PFA) for 20 min at room temperature (r.t.), and permeabilized in 0.5% Tween 20 for 20 min at 37°C. Primary Abs and AF-conjugated secondary Abs were incubated for 60 min at 37°C, the excess of Abs was washed three times with T-TBS, and samples were embedded in Mowiol (Fluka Chemicals, Selzee, Germany)-DABCO (Sigma Chemical Co, Steinheim, Germany) in PBS containing 50% glycerol. The samples were analysed by Leica DMI8 inverted confocal microscope (confocal part: TCS SP8; Leica Microsystems GmbH, Wetzlar, Germany), equipped with HC PLAPO CS2 objective (×631.40 oil), four lasers (UV with Diode 405 for DAPI; Ar 488 for AF^488^; DPSS 561 for AF^555^ and AF^595^; and He/Ne 633 for AF^680^), and four detectors (two of which are PMT and two are HyD). Images were acquired in sequential mode (515 × 515 pixels, z-series of 0.5 μm) using LAS (Leica Application Suite) X version 3.5.6.21594 software (Leica Microsystems GmbH, Wetzlar, Germany). The offset was set to 0%–1.5% depending on the background. All samples that were compared within an experiment were imaged with the same parameters.

### 2.5 Time-lapse acquisitions using holotomographic microscopy

Holotomographic microscopy in combination with epifluorescence was performed using the 3D Cell-Explorer Fluo (Nanolive, Ecublens, Switzerland) with a ×60 air objective at a wavelength of λ = 520 nm. The irradiance of the laser was 0.2 nW/μm2, and the acquisition time per image was 45 m. An top-stage incubator (Oko-lab, Pozzuoli, Italy) equipped with a heatable glass lid to avoid condensation was used to maintain physiological conditions during live cell imaging, including a constant temperature of 37°C, 90% relative humidity and 5% CO_2_ concentration. For imaging, 50,000 cells infected with Δm138-MCMV (MOI 10) were plated in 35-mm glass-bottomed Ibidi dishes (Ibidi GmbH, Germany). The dishes were placed in the incubation chamber for acclimatization prior to imaging. Imaging started 6 h after infection. The refractive index image was acquired every 2.5 min for 11 h in combination with epifluorescence every second frame. The time-lapse images were saved as 3D stacks using STEVE software (Nanolive SA, Ecublens, Switzerland), which controls the 3D Cell Explorer microscope. The 3D volumes were converted to 2D maximal projections along the *z*-axis. The 3D stacks obtained with DHTM were digitally stained with STEVE, divided into voxel segments and exported as TIFF files.

### 2.6 Image analysis

Pericentriolar (pc) and perinuclear (pn) accumulation of Rab10 fluorescence signal in MCMV-infected cells within an angle of α ≤ 90° was defined as pre-AC as previously described (Štimac et al., 2021). The pre-AC-positive cells were counted on at least 10 fields of view using an Olympus BX51 epifluorescence microscope equipped with a DP71CCD camera (Olympus, Tokyo, Japan) and a UPlanFL N 40×/0.75 objective.

Colocalization was determined using Manders’ overlap coefficients (M1 and M2) calculated on the entire z-stack of confocal images (at least 8–12 slices; 120.37 × 120.57 nm pixel size) using the Fiji-ImageJ and the JACoP plugin (https://imagej.net/ij/plugins/track/jacop2.html, accessed on 25 October 2024) (Bolte and Cordelières, 2006). After splitting the red, green and blue channels and subtracting the background, the colocalization of the pixels between two selected channels was determined as previously described (Marcelić et al., 2022).

The DHTM images exported in TIFF format from STEVE were processed in Fiji-ImageJ. The overlaid images with RI and fluorescence signals were split, the cell area and the fluorescence area above the background were delineated with the freehand tool and used for measurement, including the surface area and average fluorescence intensity. The cell area was calculated on the RI image through the focal plane and the Rab10-positive area was calculated on the fluorescence image using the freehand tool and the region of interest (ROI) manager. The average fluorescence intensity of the Rab10-positive area was calculated on the fluorescence image using the ROI manager.

### 2.7 siRNA silencing

Small interfering (si)RNA sequences were acquired as follows: negative (control) siRNA (1022076) was from Qiagen (Hilden, Germany); siRNA for Rab3IP/Rabin8 (Sc-152666) and siRNA for EHBP1 (Sc-144602) were from Santa Cruz Biotechnology Inc. (Dallas, United States); and siRNA for Rab10 (L-040862-01–0005) was from Dharmacon Inc. (Laffayete, Colorado, United States). Briefly, siRNA and RNAiMAX Lipofectamine Reagent (Invitrogen, Carlsbad, CA, United States) were mixed according to the manufacturer’s guidelines, added dropwise to the cells, incubated at 37°C for 48 h, and analysed or proceeded to the experiment. The final concentration of siRNA for Rab3IP/Rabin8 was 60 nM, for EHBP1 was 100 nM, and for Rab10 was 30 nM.

### 2.8 Western blot

Cells were lysed in RIPA buffer containing protease inhibitors (Cat. No. 11697498001, Roche Diagnostics GmbH, Unterhaching, Germany), the lysates were mixed with the sample buffer, the proteins were separated by SDS-PAGE using Bio-Rad PowerPac (Universal, Hercules, CA, United States) and blotted onto a polyvinylidene (PVDF-P) difluoride membrane (Millipore, XXX) using the Bio-Rad Trans-Blot Turbo Transfer System (Hercules, CA, United States) at 60–70 V for 1 h. Membranes were blocked for 1–2 h in 1% blocking reagent (Roche Diagnostics GmbH, Mannheim, Germany), incubated overnight at 4°C with primary Abs, washed three times in T-TBS (TBS with 0.05% Tween 20; pH = 7.5) and incubated for 45–60 min with peroxidase (POD)-conjugated secondary Abs in T-TBS with 0.5% blocking reagent. After washing in T-TBS, the signal was detected by chemiluminescence (SignalFire [TM] Plus ECL Reagent or SignalFire [TM] Elite ECL Reagent; Cell Signaling, Cat. No. 12630S and 12757P, respectively) using Transilluminator Alliance 4.7 (Uvitec Ltd., Cambridge, United Kingdom) and ImageQuant LAS 500 (GE Healthcare Bio-Sciences AB, Upsala, Sweden). In each experiment, β-actin was used as a loading control and detected on the same membrane. The chemiluminescence signal was quantitatively analyzed using ImageQuantTL (version 10.2., Cytiva) and ImageJ 1.53 software. All values were normalized to the signal of β-actin by calculating the lane normalization factor as follows: observed actin signal for each lane/highest observed actin signal for the blot. The value normalized to actin was then used to normalize the experimental signal (raw signal value/normalized actin index). The kinetics of host cell protein expression during MCMV infection was calculated as = normalized experimental signal (t_x_ hpi)/normalized experimental signal (t_0_ hpi).

### 2.9 Subcloning of EGFP-Rab10_wt_ and EGFP-Rab10_Q68L_ into the lentiviral vector pLIX-Kan_PstI

EGFP-Rab10_wt_ and EGFP-Rab10_Q68L_ ORFs were subcloned from EGFP-Rab10 (Rzomp et al., 2003) and EGFP-Rab10-Q68L (Huang et al., 2010) plasmids (a gift from dr. Marci Scidmore; Addgene plasmids # 49742 and # 49544) into the pLIX402_Kan lentiviral vector with doxycycline-inducible expression of transgene derived from pLIX402 (Addgene plasmid # 41394) by insertion of Kanamycine kassete (a gift from dr. Martin Messerle (Hannover, Germany) (Kutle et al., 2020). Briefly, the primers for PCR amplification of bothORFs (F-EGFP-Rab10: 5′-TTT TTT CTG CAG ATG GTG AGC AAG GGC GAG-3′, and R-GFP-Rab10: 5′-TTT TTT GGA TCC TCA GCA TTT GCT CTT CC-3′) were created according to original plasmids. At the same time, the pLIX-Kan vector was cleaved with PstI (cat. no. R3140S, New England Biolabs Inc.) and BamH1 (cat. no. R3136S, New England Biolabs Inc.) restriction endonucleases. Finally, rapid DNA ligation kit (cat. no. K1422, Thermo Fisher Scientific, Waltham, MA, United States) was used to ligate EGFP-Rab10 insert into pLIX lentiviral vector (without Kan cassette).

The verified pLIX EGFP-Rab10_wt_ and EGFP-Rab10_Q68L_ plasmids were used for production of lentiviruses in HEK 293T cells (ATCC clone A31, ATCC CRL-3216, Manassas, VA, United States). Transduction of NIH 3T3 cells and selection of NIH 3T3 EGFP-Rab10_wt_ and NIH 3T3 EGFP-Rab10_Q68L_ cells with puromycin (2.5 μg/mL) is described in Section 2.11.

### 2.10 Construction of the pGenLenti Rab10-BioID2-HA plasmid

Rab10-(GGGGS)_13_-mBioID2-HA fusion sequence was designed *in silico* by in-frame joining of mouse Rab10 protein-coding sequence (retrieved from Ensembl genome database, ensesmbl. org), 13×(GGGGS) linker sequence (Kim et al., 2016), mouse codon-optimized BioID2 (mBioID2) sequence, and a sequence encoding HA-tag. BioID2 sequence was retrieved from the plasmid MCS-BioID2-HA (a kind gift from Kyle Roux, n2t.net/addgene:74224), and subsequently codon-optimized for expression in mouse cells using GenSmart™ Codon Optimization tool available at www.genscript.com/gensmart-free-gene-codon-optimization.html). Once designed, the Rab10-(GGGGS)_13_-mBioID2-HA fusion sequence (see supplementary material and methods) was submitted to GenScript, where the corresponding DNA fragment was synthesized and subcloned into the pGenLenti lentiviral vector (GenScript Biotech., New Jersey, United States) between EcoRI and BamHI restriction sites, giving rise to the pGenLenti Rab10-BioID2-HA plasmid. Purified pGenLenti Rab10-BioID2-HA was then used to produce lentiviral particles in HEK 3T3 cells (ATCC clone A31, ATCC CRL-3216, Manassas, VA, United States). Obtained lentiviral particles carrying Rab10-BioID2-HA were then used to transduce NIH 3T3 cells, and Rab10-BioID2 positive NIH 3T3 cells were selected using puromycin (2 μg/mL).

### 2.11 Generation of NIH 3T3 EGFP-Rab10_wt_, NIH 3T3 EGFP-Rab10_Q68L_ and NIH 3T3 Rab10-BioID2-HA cell lines

NIH 3T3 cells expressing EGFP-Rab10 or Rab10-BioID2-HA fusion proteins were generated by transduction with lentiviruses. 5 μg of the lentiviral plasmid pLIX EGFP-Rab10_wt_, pLIX EGFP-Rab10_Q68L_ or pGenLenti Rab10-BioID2-HA were mixed with 10 μg of p8.91 (a gift from Simon Davis; Addgene plasmid # 187441; http://n2t.net/addgene:187441; RRID:Addgene_187441) (Sušac et al., 2022) and 0.5 μg of plasmid p-CMV-VSV-G (a gift from Bob Weinberg; Addgene plasmid #8454; http://n2t.net/addgene:8454; RRID:Addgene_8454) (Stewart et al., 2003), dissolved in 1.5 mL Optimem (Thermo Fisher Scientific, Waltham, MA, United States; (Cat. No. 31-985-070). The solution was mixed with 41 μL Lipofectamine 3000 in 1.5 mL Optimem and carefully added to 80%–85% confluent HEK 393T cells (ATCC clone A31, ATCC CRL-3216, Manassas, VA, United States) cultured in 10% FCS DMEM without antibiotic. The supernatants were collected 24, 30 and 48 h after transfection. After centrifugation (5 min at 2,000 rpm) and filtration (0.45 μM filter), the lentiviruses were used to transfect NIH 3T3 cells. Puromycin (2.5 μg/mL) was used to select the fusion protein-expressing cells. The puromycin-resistant EGFP-Rab10-expressing cells were additionally sorted using the FACSAria cell sorter (Becton Dickinson and Co, San Jose, CA, United States).

### 2.12 Biotin labelling of NIH 3T3 Rab10-BioID2-HA cells and immunoprecipitation of biotinylated proteins

NIH 3T3 Rab10-BioID2-HA cells were cultured in 6 cm Petri dishes in cell culture medium with biotin (50 μM) as follows: (1) 18 h for uninfected cells; (2) 12 h before infection and 6 h together with MCMV infection (without replacement of the culture medium), and (3) 18 h together with MCMV infection. After three washes with PBS to remove excess biotin, 20% of cells were lysed in 20 μL of RIPA to achieve whole cell lysate (WCL), and 80% of the cells were lysed in 350 μL of RIPA for overnight precipitation of biotinylated proteins with NeutrAvidin Agarose (NA-A) (Thermo Fisher Scientific, SAD) at 4°C. The samples were centrifuged (14000 rpm, 1 min), and the pellet was twice washed in washing buffer 1 (1% SDS), then once in washing buffer 2 (0.1% deoxycholic acid, 1% Triton-X100, 1 mM EDTA, 500 mM NaCl, 50 mM HEPES), and washing buffer 3 (0.5% deoxycholic acid, 0.5% NP-40, 1 mM EDTA, 250 mM LiCl, 10 mM Tris-HCl pH 7.4), as described (Sakai et al., 2019; Nguyen-Tien et al., 2022). The biotinylated proteins were eluted for 10 min at 95°C in a buffer containing 10% 2-mercaptoethanol, 0.125 M Tris-HCl (pH 6.8), 0.1% SDS (w/v), 0.1% bromophenol blue (w/v), and 30% glycerol (v/v) followed by Western blot analysis together with WCL. The pIE1 served as control of infection, and β-actin as loading control.

### 2.13 Transient transfection of NIH 3T3 and Balb 3T3 cellswith EGFP-Rab10, EGFP-Rab10-Q68L, and EGFP-PH-PLC-δ1 MSCV plasmids

The original Addgene plasmids EGFP-Rab10 (Rzomp et al., 2003) and EGFP-Rab10-Q68L (Huang et al., 2010) were used for transfection to express the wild-type and GTP-locked forms of Rab10, respectively. Murine Stem Cell retroviral vector (MSCV) containing EGFP-PH-PLC-δ1 (pleckstrin homology domain from phospholipase C delta 1) was used to express the recombinant membrane domain that binds to PI(4,5)P2 (Bertović et al., 2020). Transient transfections was performed by gently mixing solutions of 1 μg of plasmids in Optimem (Gibco/Invitrogen, Grand Island, NY, United States) with 1 μL of Lipofectamine 3000 transfection reagent (Invitrogen) according to the manufacturer’s instructions and proceeded to the experimental protocols as described in Results.

### 2.14 Statistical analysis and data presentation

Statistical significance was assessed using a two-tailed Student’s t-test. Differences were considered significant when P values were <0.05 (*P ≤ 0.05; **P < 0.01; ***P < 0.001).

## 3 Results

### 3.1 Expansion of Rab10-positive domain within the reorganized membrane system of cytomegalovirus-infected cell

**FIGURE 1:**
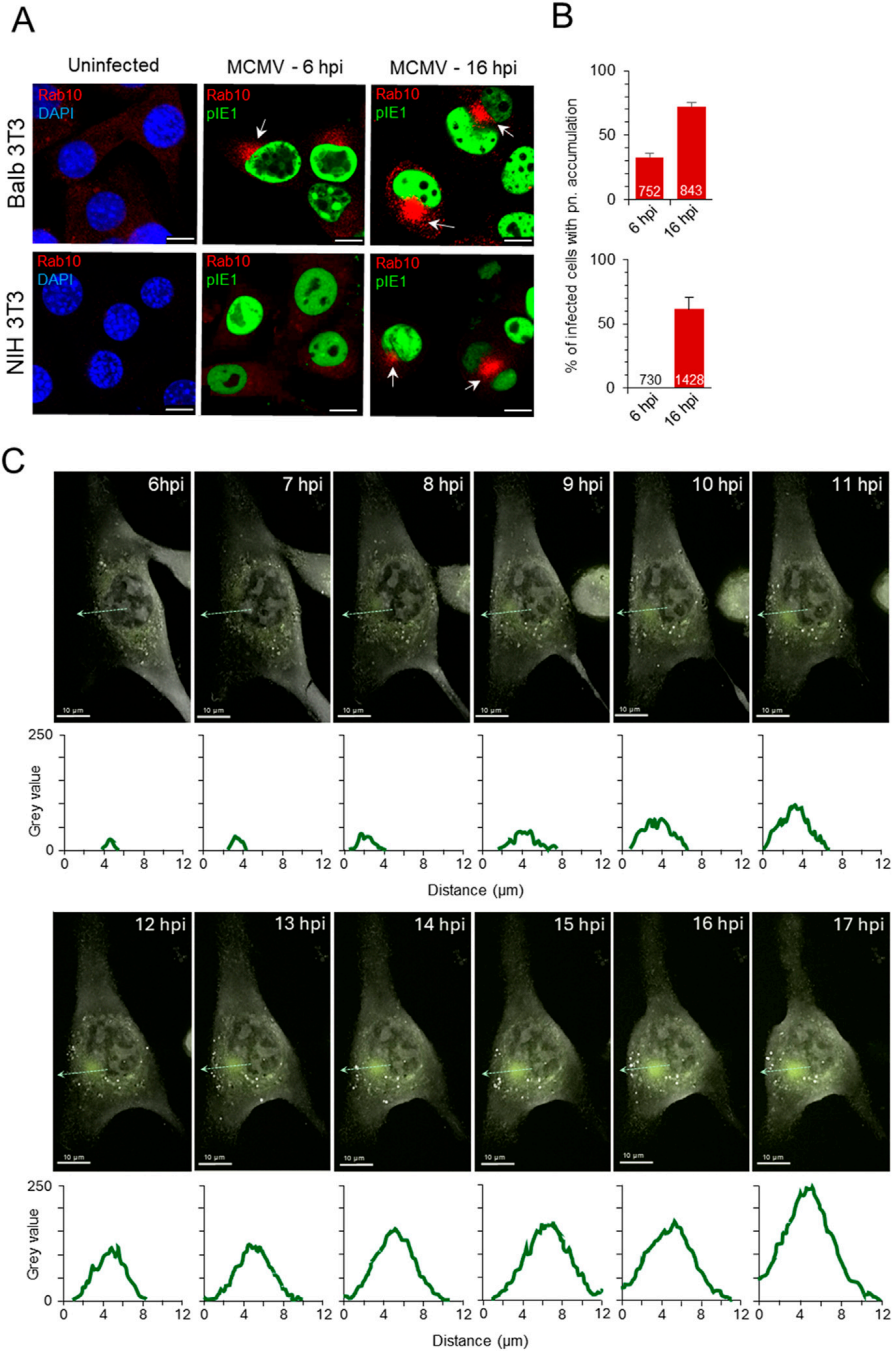
Expansion of Rab10-PD in the pre-AC of CMV-infected cells. **(A, B)** Balb 3T3 and NIH 3T3 cells, uninfected or infected with Δm138-MCMV (MOI of 10), were fixed 6 and 16 h post-infection (hpi), permeabilized and stained with Abs against Rab10 (red) in combination with Abs against pIE1 in infected cells for infection control (green) or with DAPI (blue) in uninfected cells for nuclei staining. Shown are the merged confocal images through the focal plane. The arrows show the perinuclear accumulation of Rab10 in the pre-AC. Bars, 10 μm. The percentage of cells **(B)** in three independent experiments with perinuclear (pn) Rab10 accumulation in MCMV-infected (pIE1-positive) cells is shown as mean ± SD. The numbers in the bars indicate the total number of cells analyzed. **(C)** Long-term high-frequency live cell imaging of MCMV-infected cells using DHTM with attached epifluorescence module. Expression of EGFP-Rab10 in the NIH 3T3 EGFP-Rab10 cell line was induced by doxycycline (2 μg/mL), and after 24 h, cells were infected with Δm138-MCMV (MOI of 10). Cells were imaged continuously from six hpi with DHTM at intervals of 2.5 min for refractive index (RI) and 5 min for fluorescence signal. The overlaid images show RI and fluorescence signal at 1-h intervals during the E phase of infection (6–17 hpi). The RI and fluorescence images as well as the overlaid video can be seen in the supplementary (Supplementary Video S1). The fluorescence intensity profiles along the light green dashed arrow lines are shown below the images, and the surface area and mean fluorescence intensity of the Rab10-positive perinuclear region is shown in Supplementary Figure S3. Another example of the cell is shown in Supplementary Figure S4 and Supplementary Video S2.

In our previous studies on Balb 3T3 cells (Lučin et al., 2020; Štimac et al., 2021; 2024), we reported the perinuclear accumulation of Rab10-positive membranes in Δm138-MCMV-infected cells in the E phase of infection. Very little endogenous Rab10 can be detected on membrane structures in these cells (Figure 1A, uninfected). After infection, perinuclear accumulation of Rab10 was observed by confocal imaging in 32.8% ± 2.8% of infected cells at six hpi, which progressed during the E phase of infection and resulted in 72.0% ± 3.3% of infected cells showing increased perinuclear Rab10 staining at 16 hpi, at the end of the E phase (Figures 1A, B). Nuclear accumulation of immediate early one (pIE1) was used as an indicator of infection (Figure 1A). For infection, we used recombinant MCMV with deleted m138 gene encoding the E-phase protein with FcR properties (Thäle et al., 1994) to visualize endogenous host cell proteins and avoid nonspecific binding of antibody reagents in immunofluorescence studies. Infection with the wild type virus, expressing m138 protein with Fc-binding properties (Supplementary Figure S1A), resulted in indistinguishable reorganization of the membrane system in the E phase of infection, including dislocation of the Golgi (Supplementary Figure S1A) and perinuclear accumulation or Rab10 (Supplementary Figure S1B).

Similar to Balb 3T3 cells, Rab10 was barely detected in cytoplasmic structures of uninfected NIH 3T3 cells (Figure 1A). After MCMV infection a similar proportion of cells (61.3% ± 9.3%) developed enhanced perinuclear Rab10 accumulation at 16 hpi, but no perinuclear Rab10 accumulation was observed at six hpi (Figures 1A, B). Apart from the observed delay in Rab10 accumulation, the development of pre-AC in NIH 3T3 cells is consistent with previously published data on Balb 3T3 cells (Lučin et al., 2020; Štimac et al., 2021), including the rapid turnover of activated Rab10 at endosomal membranes of the uninfected cell.

To investigate the expansion of Rab10-PD in living cells, we generated cell lines on the NIH 3T3 background with inducible expression of wild-type and GTP-locked (Q68L) mutant of Rab10 (NIH 3T3 EGFP-Rab10_wt_ and NIH 3T3 EGFP-Rab10_Q68L_, respectively), as confirmed by fluorescence imaging (Supplementary Figure S2A) and Western blot (Supplementary Figure S2B). A modest organelle-associated fluorescent signal was detected in these cells after 24 h of induction, mainly in the pericentriolar region (Supplementary Figure S2A). We infected these cells with MCMV 24 h after induction of EGFP-Rab10_wt_ expression and performed long-term time-lapse imaging during the early phase of infection. We combined digital holo tomographic microscopy (DHTM), which provides high-resolution refractive index (RI) images at high spatial resolution with ultra-low power, with epifluorescence time-lapse imaging of the fluorescence signal. Infected cells were placed into the top-stage incubator immediately after infection, and DHTM recording began 2–3 h later at high frequency (every 2.5 min for refractive index and every 5 min for fluorescence) over the next 16–17 h. Several cytopathic effects were observed during DHTM recording, including nuclear rotation as described for HCMV-infected cells (Procter et al., 2018) (Supplementary Video S1). At 6–7 hpi, nuclear indentation began in the pericentriolar region, accompanied by the concentration of fluorescence signal as a pericentriolar spot at eight hpi (Figure 1C; Supplementary Figure S3; Supplementary Video S1). As the E phase progressed (from 8 to 17 hpi), the surface area and fluorescence in the perinuclear region increased (Figures 1C; Supplementary Figure S3, S4; Supplementary Videos S1, S2), demonstrating the expansion of Rab10-PD. Similar perinuclear accumulation was observed by live imaging of MCMV-infected cells expressing GTP-locked Rab10 (Supplementary Video S3) and in cells transfected with EGFP-Rab10_Q68L_ (Supplementary Figure S5), indicating membrane recruitment of GTP-bound form of Rab10 at membranes within the pre-AC. We also confirmed EGFP-Rab10_wt_ accumulation in the E phase after infection with the wild-type MCMV (Supplementary Video S4).

### 3.2 Accumulation of EHBP1, Rabin8 and Rab10 effectors in the inner pre-AC

**FIGURE 2:**
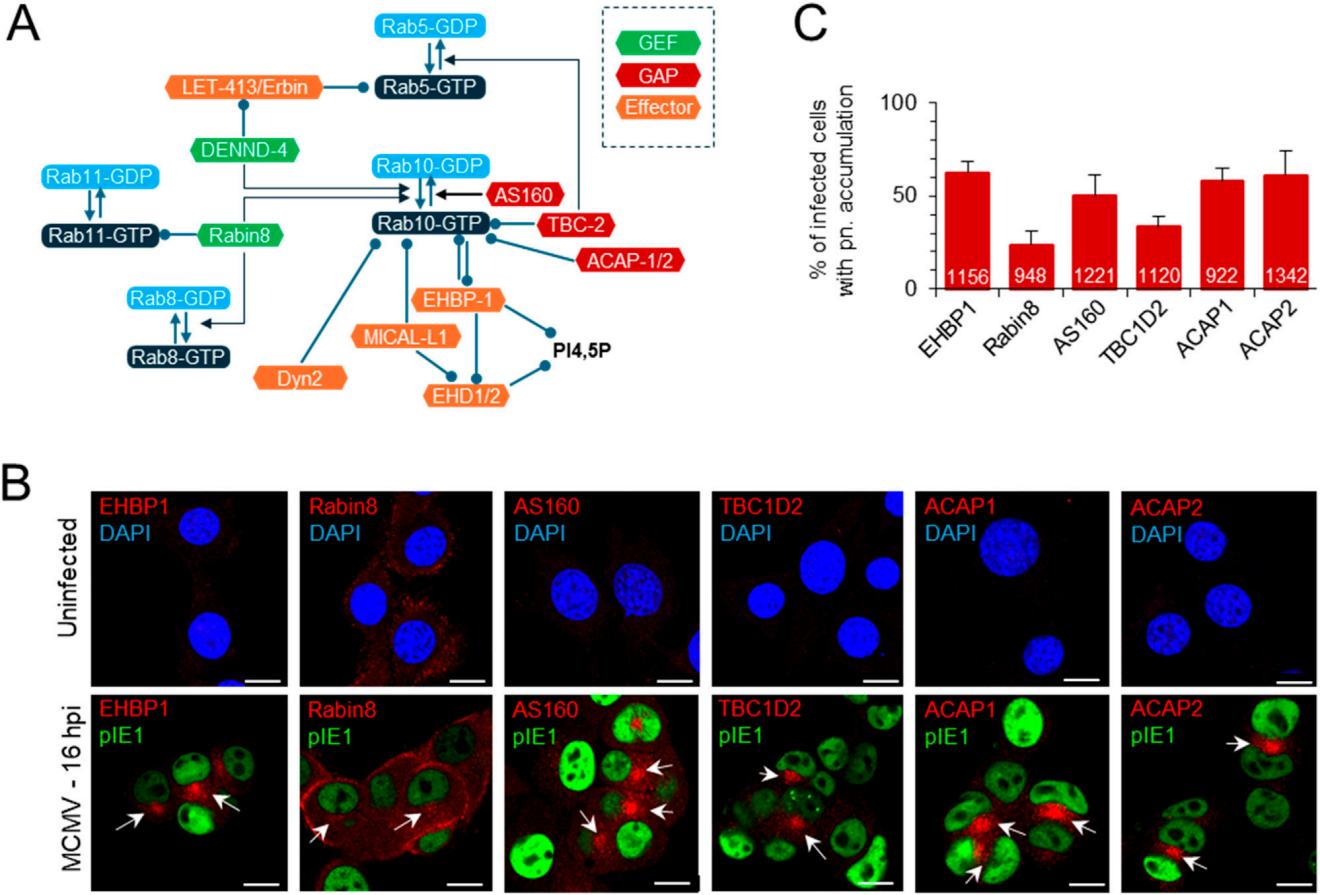
Perinuclear accumulation of EHBP1, Rabin8 and Rab10 effectors within the pre-AC. **(A)** Schematic representation of the regulatory network involving Rab10 at the EE-RE/ERC interface, Rab10 interactors and effectors. Arrows indicate activity and circles recruitment. GEF, guanine nucleotide exchange factors; GAP, GTPase-activating proteins. **(B)** Immunofluorescence analysis of EHBP1, Rabin8 and Rab10 effectors in uninfected and MCMV-infected cells. NIH 3T3 cells were infected or uninfected with Δm138-MCMV (MOI of 10) and fixed at 16 hpi, permeabilized and stained with Abs against EHBP1, Rabin8, AS160, TBC1D2, ACAP1 or ACAP2 (red) in combination with Abs against pIE1 in infected cells to control infection (green) or with DAPI (blue) in uninfected cells to stain nuclei. Shown are the merged confocal images through the focal plane of a representative experiment. The arrows indicate pericentriolar accumulation in the pre-AC. Bars, 10 μm. **(C)** Percentage of cells with perinuclear (pn) accumulation in MCMV-infected (pIE1-positive) cells, shown as mean ± SD from three independent experiments. The numbers in the bars indicate the total number of cells analyzed.

Although Rab10 can interact with many host cell proteins associated with the regulation of membrane flux in the secretory and endosomal system of the cell (Chua and Tang, 2018; Zhu et al., 2024), its perinuclear accumulation in CMV-infected cells suggests membrane expansion at the EE-RE/ERC-TGN interface (Lučin et al., 2020). Therefore, we performed immunofluorescence analysis of the known interactors of Rab10 at EE and the ERC (Figure 2A). As previously reported in Balb 3T3 cells (Lučin et al., 2020), we observed perinuclear accumulation of several small GTPases that act at the EE-ERC interface and are associated with Rab10 activation, including Rab5 and Rab11. Similar was observed in NIH 3T3 cells (data not shown). As described for Rab10, six of the Rab10 interactors analyzed in uninfected NIH 3T3 cells showed no staining of membrane organelles, with the exception of Rabin8, which was detected on various structures at the cell periphery (Figure 2B). In MCMV-infected NIH 3T3 cells, endogenous EHBP1, a protein essential for the recruitment of Rab10 to EEs (Farmer et al., 2021), accumulated in the perinuclear area at 16 hpi (Figure 2B) in 62.4% ± 6.1% of infected cells (Figure 2C), similar to Rab10 (Figure 1B). Endogenous Rabin8, an effector of Rab11 that functions as a GEF for Rab10 and Rab8 downstream in the ERC (Homma and Fukuda, 2016), accumulated mainly at the cell periphery and to some extent in the perinuclear area (Figure 2B) of 23.8% ± 7.3% of infected cells at 16 hpi (Figure 2C). The Rab10 effectors TBC1D2 (GAP for Rab5 (Liu and Grant, 2015), and AS160 (known as TBC1D4; GAP for Rab10 (Homma et al., 2021)) showed perinuclear accumulation (Figure 2B) in 33.3% ± 6.0% and 50% ± 11.6% of infected cells, respectively (Figure 2C). ACAP1 and ACAP2, which act as GAPs for ARF6 downstream of Rab10 (Shi and Grant, 2013) accumulated perinuclearly (Figure 2B) in a similar fraction of cells as Rab10 (58.1% ± 7.1% and 61.1% ± 12.9% of infected cells, respectively; Figure 2C). Unfortunately, we were unable to accurately monitor DENND4, the Rab5-dependent GEF for Rab10 (Yoshimura et al., 2010), and MICAL-L1, the Rab10 effector associated with tubulation (Farmer et al., 2021), with the available antibody reagents. Essentially identical results were obtained on Balb 3T3 cells at 16 hpi, and some of these Rab10 effectors accumulated to a similar extent as Rab10 at six hpi (Supplementary Figure S6). Colocalization analysis of endogenous EHBP1 and Rabin8 was performed on NIH 3T3 GFP-Rab10 cells after 24 h induction of EGFP-Rab10 expression, as all antibody reagents were of rabbit and simultaneous staining with endogenous Rab10 was not possible. As expected, perinuclearly accumulated Rab10 colocalized with EHBP1 and Rabin8 (Supplementary Figure S7). These data show perinuclear accumulation of Rab10 interactors supporting the conclusion of expansion of Rab10-PD in the area of inner pre-AC.

The accumulation of Rab10 and its known interactors at EE-RE/ERC is not associated with increased transcriptional activity (Supplementary Figure S8), as shown by the analysis of our previously published transcriptome (Lučin et al., 2020) and total protein accumulation, as shown by Western blot analysis of Rab10, EHBP1, and Rabin8 in Balb 3T3 cells (Supplementary Figure S6C). Thus, the expansion of Rab10-PD is associated with prolonged retention of Rab10 and its interactors at membranes within the inner pre-AC.

### 3.3 Rab10 interacts with EHBP1 and MICAL-L1 in MCMV infected cells

**FIGURE 3:**
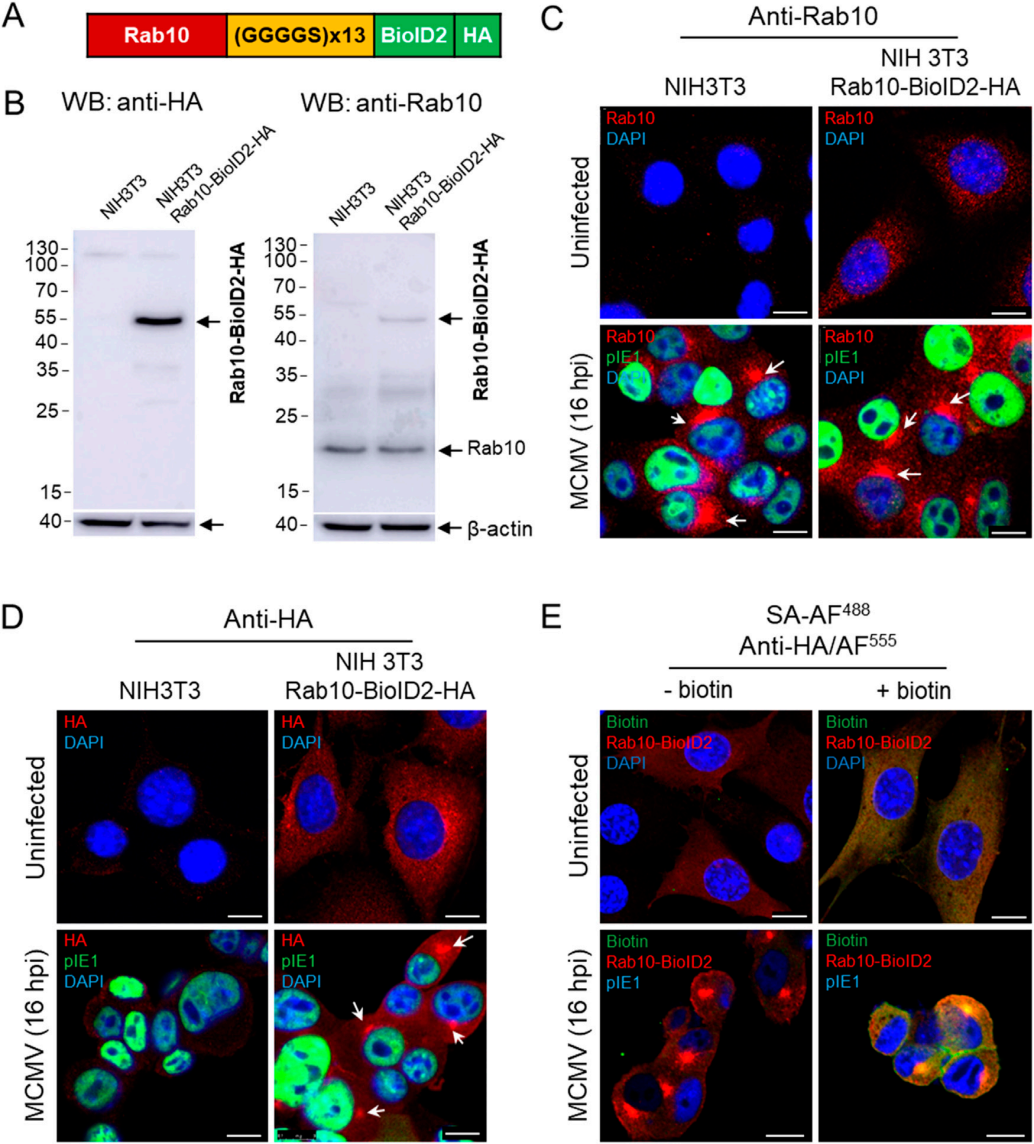
Establishment of the NIH 3T3 Rab10-BioID2-HA cell line. The Rab10-(GGGGS)13-mBioID2-HA fusion sequence **(A)** subcloned into the pGenLenti lentiviral vector was used to establish the NIH 3T3 Rab10-BioID2-HA cell line, which was analyzed by Western blot **(B)** for the expression of Rab10-BioID2-HA using antibodies against HA and Rab10. **(C, D)** Immunofluorescence analysis of uninfected and MCMV-infected (Δm138-MCMV at an MOI of 10 for 16 h) paternal and NIH 3T3 Rab10-BioID2-HA cells stained with antibodies against Rab10 and HA (red fluorescence). DAPI was used to stain the nucleus and anti-pIE1 (green fluorescence) to visualize the infected cells. Shown are the merged confocal images through the focal plane of a representative experiment. Arrows show the perinuclear accumulation of Rab10 and Rab10-BioID2-HA in paternal and NIH 3T3 Rab10-BioID2-HA. **(E)** Immunofluorescence analysis of the biotinylation reaction in NIH 3T3 Rab10-BioID2-HA cells treated with biotin. Uninfected and MCMV-infected (Δm138-MCMV at an MOI of 10) cells were treated with biotin (50 μM) and after 18 h fixed, permeabilized and stained with AF^488^-conjugated streptavidin (SA), anti-HA and anti-IE1, followed by AF^555^- and AF^680^-conjugated non-cross-reactive secondary antibodies, respectively. The complete set of images is shown in Supplementary Figure S9. Bars, 10 μm.

The accumulation of Rab10 and its interactors at the membranes of the inner pre-AC suggests Rab10-based recruitment and retention and should be detectable by a biochemical method to identify protein interactions. To test this hypothesis, we used the proximity-dependent biotin identification (BioID) assay (Roux et al., 2012; Sakai et al., 2019) to identify proteins in close proximity (10–20 nm) to Rab10. We fused Rab10 with BioID2 and HA and generated an NIH 3T3 Rab10-BioID2-HA cell line expressing Rab10-BioID2-HA fusion protein with an expected molecular weight of 54.2 kDa (Figures 3A, B). In this cell line, Rab10-BioID2-HA was visualized in the cytosol in membrane structures that exhibited vesicular and tubular patterns (Figures 3C, D). After MCMV infection, Rab10-BioID2-HA concentrated in the perinuclear region with similar kinetics as in paternal NIH 3T3 cells, as shown by immunofluorescence visualization with anti-Rab10 and anti-HA antibodies (Figures 3C, D). To investigate whether the biotin ligation response occurs at the sites of Rab10 accumulation, we exposed MCMV-infected cells to biotin for 18 h during the early phase of infection. Staining with streptavidin-Alexa-Fluor 488 (SA-AF488) revealed an accumulation of biotinylated proteins in the pre-AC that strongly overlapped with areas expressing Rab10-BioID2-HA, as shown by staining with anti-HA antibodies (Figure 3E; Supplementary Figure S9).

**FIGURE 4:**
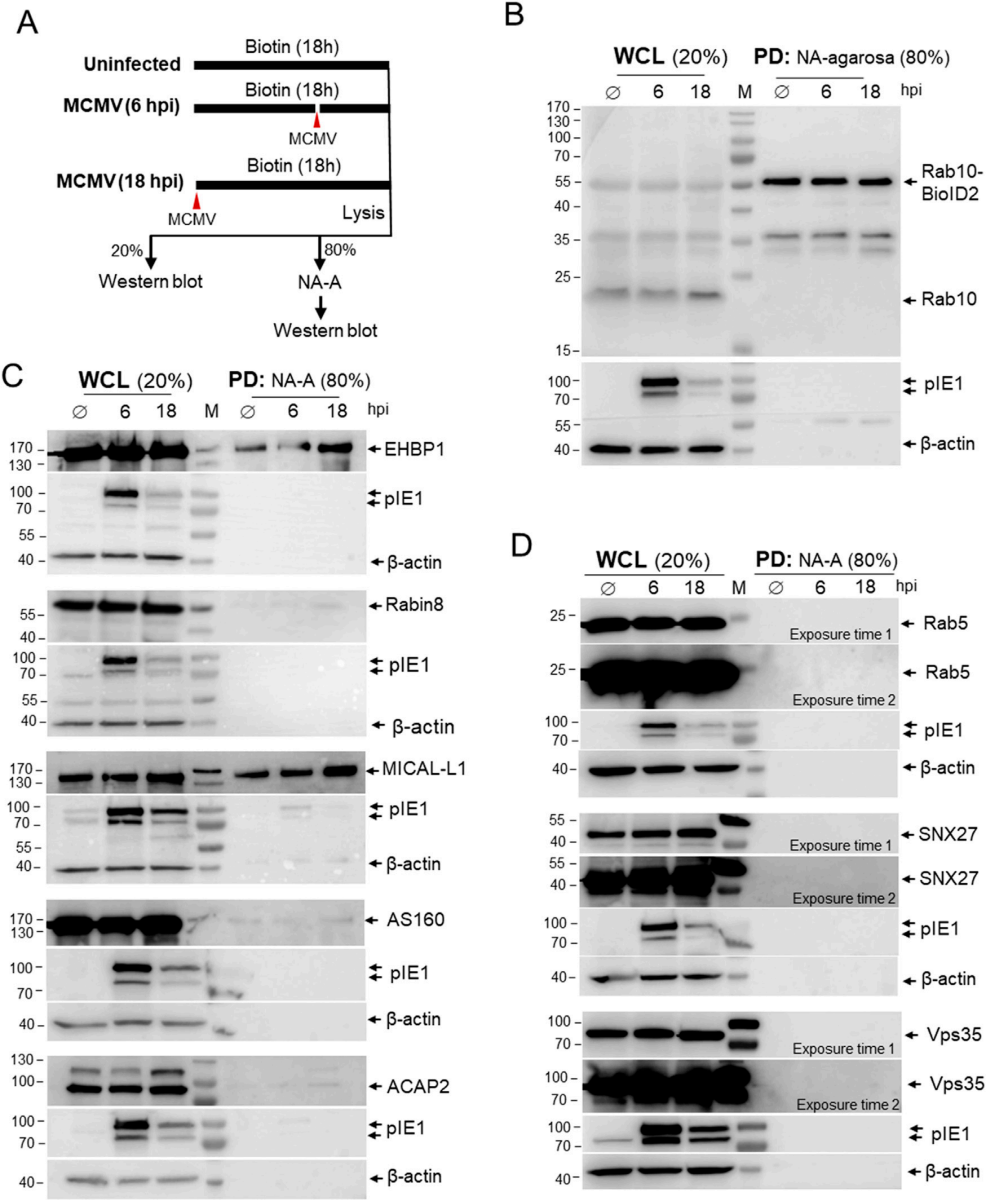
Proximity-dependent biotin identification (BioID) of Rab10 interactors in MCMV-infected cells. **(A)** *Schematic representation of the BioID assay*. NIH 3T3 Rab10-BioID2-HA cells were exposed to biotin and left uninfected (Ø) for 18 h or infected with Δm138-MCMV (MOI of 10) at one time point or 12 h after biotin and incubated in biotin for a total of 18 h to reach 18 and 6 h post-infection, respectively. Cell samples were lysed and 20% of the sample was used for Western analysis as whole cell lysate (WCL) and 80% of the sample was used for pull-down (PD) with NeutraAvidin-Agarose (NA-A) and subsequent Western blot analysis. The membranes with WCL and NA-A pull-down (PD) samples were analyzed with **(B)** antibodies against Rab10 and **(C)** antibodies against Rab10 interactors: EHBP1, Rabin8, MICAL-L1, AS160 and ACAP2; and **(D)** non-interacting cellular proteins Rab5, SNX27 and Vps35. Expression of pIE1 and β-actin in each sample served as infection and loading controls, respectively, and was performed on the same membranes. Shown are representative blots from four (EHBP1 and MICAL-L1) and two (Rabin8, AS160, ACAP2, Rab5, SNX27 and Vps35) experiments. Original raw blots and unedited ECL images are shown in supplementary (Supplementary Figure S10).

To identify proteins that interact with Rab10, we exposed Rab10-BioID2-HA-expressing cells to biotin for 18 h (Figure 4A). Cells were uninfected (Ø), infected together with the addition of biotin, or infected after 12 h in the biotin-containing medium. After 18 h, uninfected, six hpi, and 18 hpi samples were lysed and the lysates were precipitated with NeutrAvidin (NA) agarose to pull-down (PD) biotinylated proteins and analyzed by Western blot. To visualize the proteins that were in close contact with Rab10-BioID2-HA, the membranes were stained with antibodies against Rab10 (Figure 4B), the Rab10 interactors (Figure 4C), and three control proteins that are functionally related to Rab10 at the EE-RE/ERC interface but are not expected to establish close contact with Rab10 (Figure 4D). The whole cell lysates (WCL) served as a control to show the endogenous levels of these proteins (Figures 4B–D). Detection of MCMV protein pIE1 and β-actin served to identify the level of infection and loading control, respectively, at the same membrane as biotinylated host cell proteins (Figures 4B–D).

As expected, precipitation with NA agarose (PD) showed that biotinylated Rab10-BioID2-HA were expressed at similar levels in uninfected and MCMV-infected cells at six hpi and 18 hpi (Figure 4B). Although Rab5, SNX27, and Vps35 are recruited to the perinuclear region and are functionally linked to Rab10 accumulation (Lučin et al., 2020; Štimac et al., 2024), these proteins were not biotinylated in MCMV-infected Rab10-BioID2-HA-expressing cells (Figure 4D), suggesting that Rab10-PD does not involve interaction with these proteins and that there is no biotinylation after cell lysis or during immunoprecipitation. On the other hand, the increasing amounts of EHBP-1 and MICAL-L1 (Figure 4C) were precipitated in both uninfected and MCMV-infected cells at 6 and 18 hpi with NA agarose, indicating that Rab10-BioID2-HA generates similar interactions as in uninfected cells and that these interactions increase as the E phase of infection progresses. Biotinylation of MICAL-L1 and EHBP-1 also indicates that Rab10 was mobilized to TREs, as both MICAL-L1 and EHBP-1 are known TRE effectors (Sharma et al., 2009; Etoh and Fukuda, 2019; Farmer et al., 2021). In contrast, little interaction of Rab10-BioID2-HA with Rabin8 was observed at 16 hpi (Figure 4C), suggesting that very little Rab10 was recruited to TREs downstream of Rab11. In addition, very little interaction with AS160 and ACAP2 was identified (Figure 4C), suggesting that the abundant recruitment of Rab10 to TREs in MCMV-infected cells does not involve recruitment of these downstream Rab10 interactors. Thus, the pull-down detection of sustained interactions of Rab10 with EHBP1 and MICAL-L1 in MCMV-infected cells provides biochemical evidence for the expansion of Rab10-PD at EE-derived tubular endosomes, prior to or independent of the Rab11-dependent pathway of their formation.

### 3.4 Depletion of EHBP1 but not Rabin8 inhibits expansion of Rab10-PD in the early phase of infection

**FIGURE 5:**
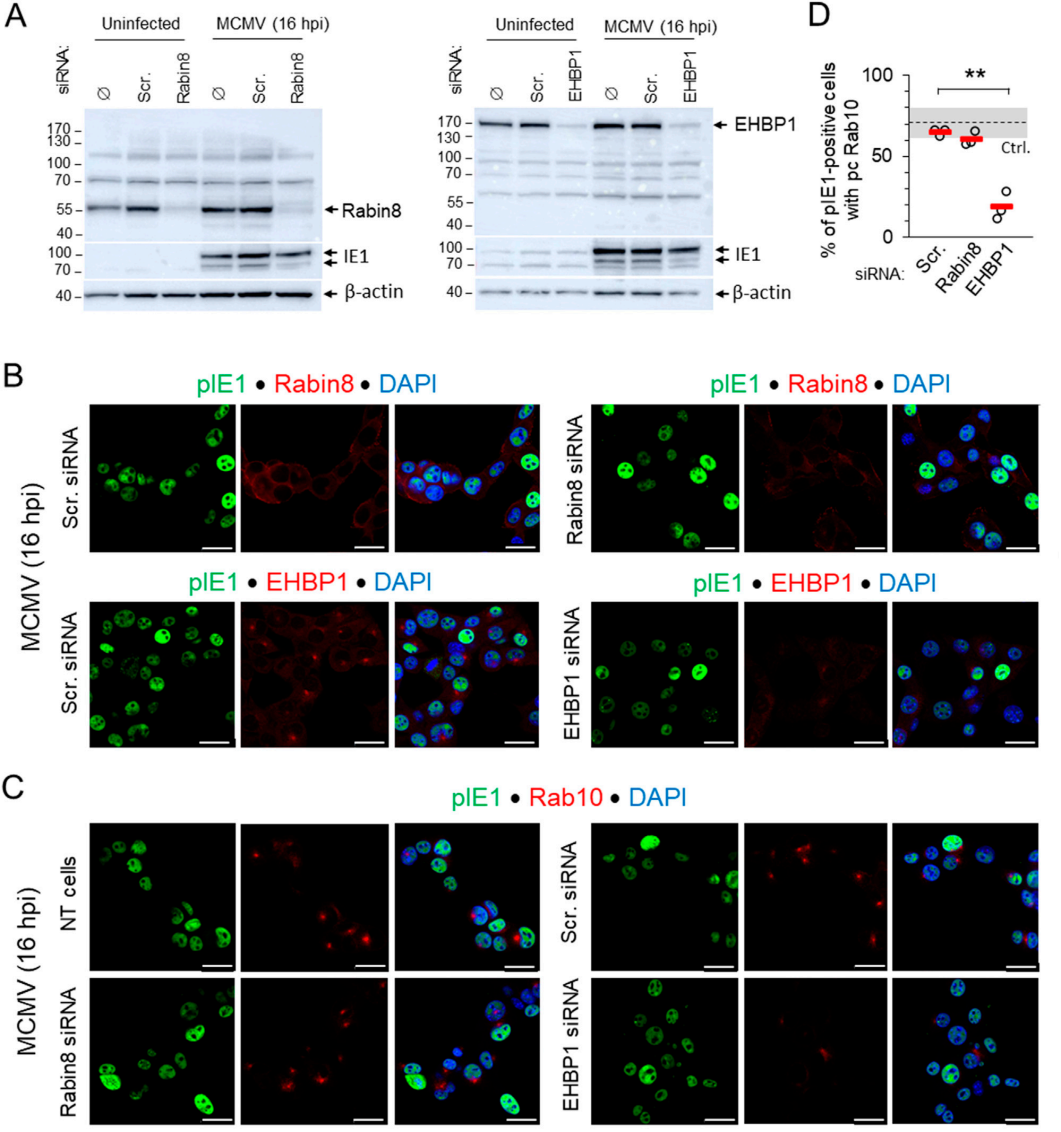
Depletion of EHBP1, but not Rabin8, prevents perinuclear expansion of Rab10-PD in pre-AC. **(A)** NIH 3T3 cells were transfected with siRNA for EHBP1 or Rabin8. Control cells were either not transfected (Ø), or transfected with “scrambled” siRNA. After 48 h, cells were infected with Δm138-MCMV (MOI of 10) for 16 h, or left uninfected, and lysed for Western blot analysis. Expression of EHBP1 and Rabin8 was determined on the same membrane along with pIE1 as a control for infection and β-actin as a loading control. **(B)** Immunofluorescence detection of endogenous EHBP1 and Rabin8 (red) in Scr. siRNA- and EHBP- or Rabin8-siRNA-treated cells at 16 h post-infection. Staining for pIE1 (green) served as a control for infection and DAPI for visualization of nuclei. Shown are the confocal images through the focal plane. **(C)** Immunofluorescence detection of endogenous Rab10 (red) in untreated (NT), Scr. siRNA- and EHBP1 or Rabin8 siRNA-treated cells. Staining for pIE1 (green) served as a control for infection and DAPI for visualization of nuclei. Shown are the confocal images through the focal plane. Bars, 25 μm. **(D)** Percentage of MCMV-infected (pIE1-positive) cells with perinuclear accumulation of Rab10 in Scr. siRNA- and EHBP1 or Rabin8 siRNA-treated cells 16 h post-infection, shown as mean ± SD. The total number of cells analysed: Scr 1,659; EHBP1 1,578; Rabin8 1,578. Ctrl., control level. Statistical significance was determined by Student’s t-test (**p < 0.01).

Since EHBP1 and Rabin8 are known Rab10 interactors required for Rab10 mobilization to membranes (Homma and Fukuda, 2016; Rai et al., 2020; Farmer et al., 2021), we observed the development of perinuclear Rab10 accumulation in MCMV-infected cells after knocking them down with siRNA. Knockdown was verified by Western blot (Figure 5A) and by immunofluorescence detection of endogenous proteins with polyclonal antibodies (Figure 5B) after MCMV infection, as endogenous staining of uninfected cells gave only a weak signal. Depletion of EHBP1 reduced the perinuclear accumulation of EHBP1 and consequently the expansion of Rab10-PD, whereas treatment with scr-RNA and depletion of Rabin8 resulted in perinuclear accumulation of Rab10-PD as in non-transfected cells (Figures 5C, D; Supplementary Table S1). The inhibitory effect of EHBP1 depletion was also observed in Balb 3T3 cells (Supplementary Figure S11). These data indicate that EHBP-1, but not Rabin8, is required for Rab10 recruitment and Rab10-PD expansion, supporting our observation that expansion does not occur downstream of Rab11-mediated recruitment of Rabin eight to the ERC, at membranes of Rab11-REs (Homma and Fukuda, 2016).

### 3.5 Saturation of PI(4,5)P_2_ inhibits expansion of Rab10/EHBP1-positive domains within the pre-AC

**FIGURE 6:**
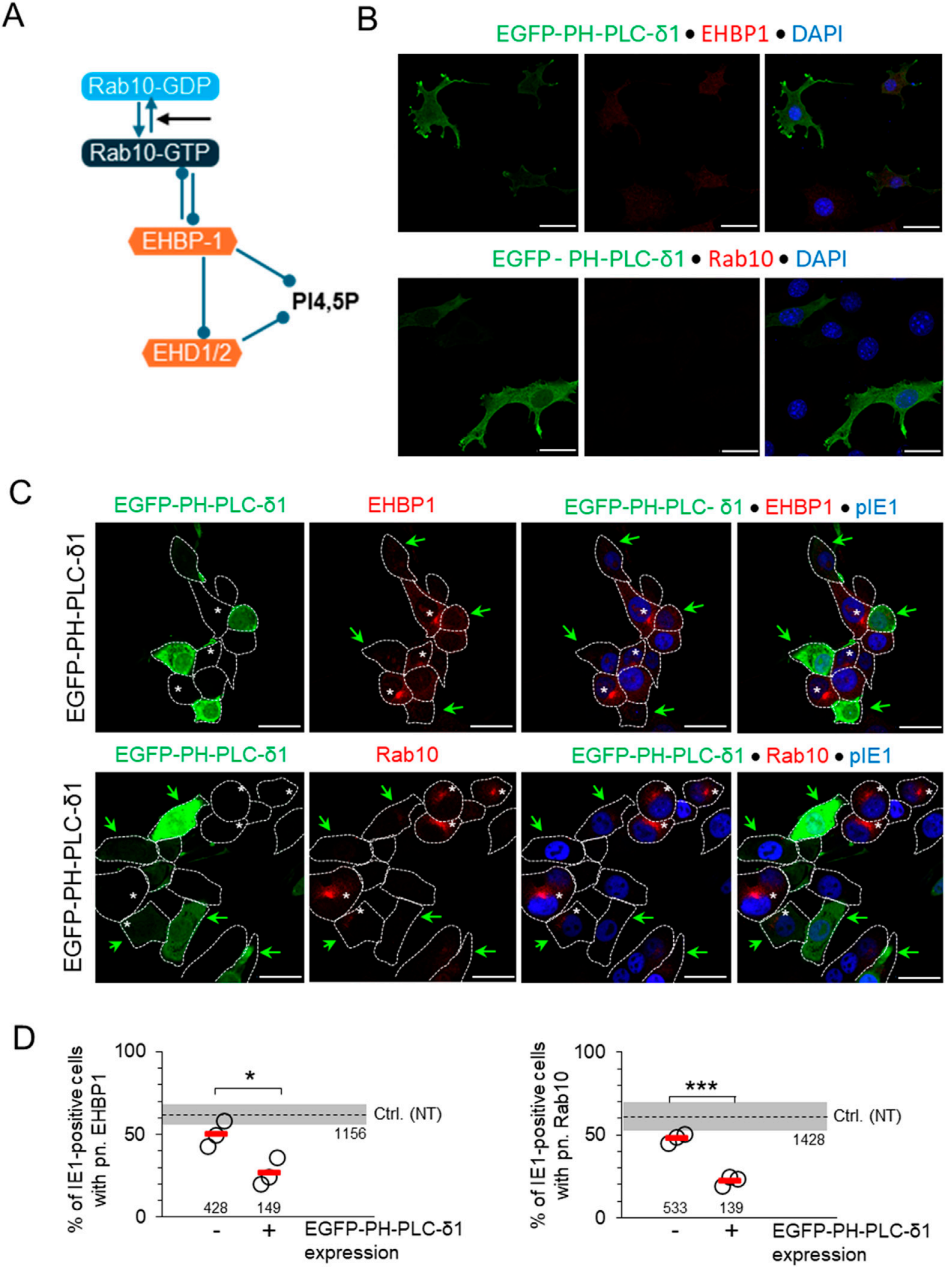
Saturation of PI(4,5)P2 domains by (over)expression of EGFP-PH-PLC-δ1 inhibits membrane recruitment of EHBP1 and expansion of Rab10-PD. **(A)** Schematic representation of the interactions of Rab10 and EHBP1 at PI(4,5)P2 endosomal domains. **(B)** NIH 3T3 cells were transfected with the MSCV expressing EGFP-PH-PLC-δ1 construct and analyzed for expression of the construct (green) and either endogenous EHBP1 or Rab10 (red) by confocal imaging 24 h after transfection. Cell nuclei were stained with DAPI (blue). **(C)** MSCV-EGFP-PH-PLC-δ1-transfected cells (24 h post-transfection) infected with Δm138-MCMV (MOI 10) for 16 h and imaged for expression of the EGFP-PH-PLC-δ1 construct (green), endogenous EHBP1 or Rab 10 (red), and virus-encoded pIE1 (blue). Shown are the focal plane images of green, red, red with blue and all three colors in MCMV-infected cells. Green arrows indicate cells expressing EGFP-PH-PLC-δ1 and white asterisks (*) indicate cells developing perinuclear accumulation of EHBP1 or Rab10. **(D)** Percentage of MCMV-infected (IE1-positive) MSCV-EGFP-PH-PLC-δ1-treated cells non-expressing (−) or expressing (+) EGFP-PH-PLC-δ1 with perinuclear (pn.) accumulation of EHBP1 and Rab10 at 16 hpi. Shown are individual experiments (circles) and mean values (red bars). Numbers indicate the total number of cells analyzed. Ctrl. (NT), control level in non-treated cells (mean ± SD). Statistical significance was determined by Student’s t-test (*p < 0.05, ***p < 0.001).

Rab10 generates and maintains EE-derived tubular recycling endosomes (TREs) by binding to EHBP1 which is recruited to PI(4,5)P2-enriched membrane domains (Etoh and Fukuda, 2019; Farmer et al., 2021) (Figure 6A). To test whether perinuclear expansion of Rab10-PD requires the establishment of PI(4,5)P2 microdomains on membranes, we transfected cells with EGFP-PH-PLC-δ1 constructs that bind and competitively saturate PI(4,5)P2 on membranes (Tan et al., 2015). In uninfected cells, this construct mainly labels PM and peripheral membranes, a major site of PI(4,5)P2 accumulation (Tan et al., 2015), but also perinuclear membranes, possibly comprising tubular endosomal structures downstream of PI3P-rich EE domains (Figure 6B; Supplementary Figure S12A). In MCMV-infected NIH 3T3 cells, expression of EGFP-PH-PLC-δ1 was accompanied by a reduction in perinuclear accumulation of EHBP-1 and Rab10 that was inversely proportional to the level of EGFP-PH-PLC-δ1 expression (Figures 6C, D; Supplementary Table S2). The same was also observed in Balb 3T3 cells (Supplementary Figures S12B, 12C; Supplementary Table S3). These data confirm that the expansion of Rab10/EHBP1-PD in the inner pre-AC of MCMV-infected cells is associated with PI(4,5)P2 membrane microdomains downstream of the PI3P-rich membrane domains of EEs, which most likely resemble TREs.

### 3.6 Expansion of the Rab10-PD is not essential for the establishment of the pre-AC

**FIGURE 7:**
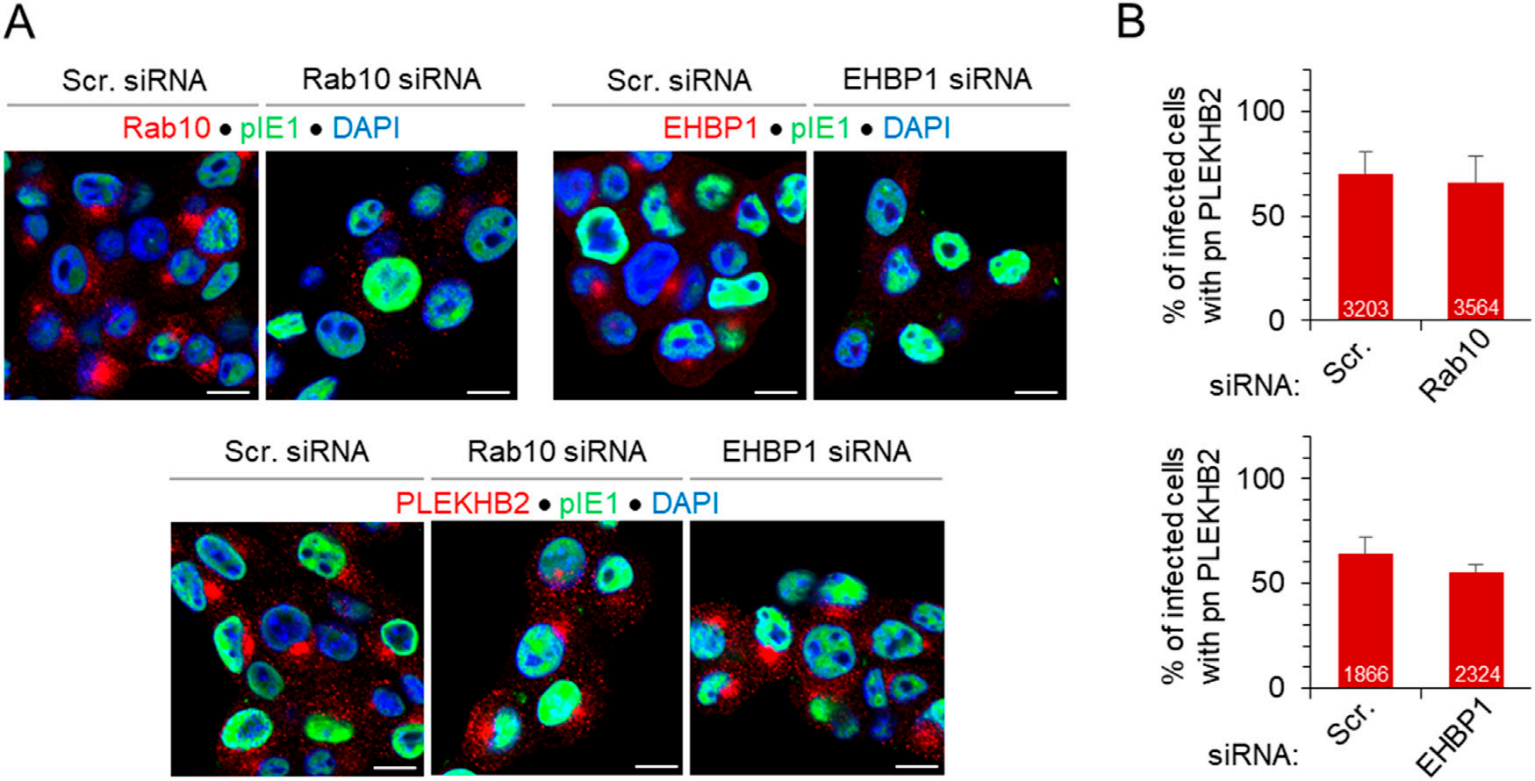
Depletion of Rab10 and EHBP1 does not prevent the establishment of pre-AC and the establishment of tubular intermediates downstream in the EE-to-ERC pathway. **(A)** Cells were transfected with either Scr, Rab10 (30 nM) or EHBP1 (100 nM) siRNA and infected with Δm138-MCMV (MOI of 10) 48 h after transfection. After 16 h, cells were fixed, permeabilized and stained with either Abs against Rab10, EHBP1 or PLEKHB2 (red fluorescence) in combination with Abs against pIE1 to control infection (green) and DAPI (blue) to stain nuclei. The merged images are shown, and the complete image sets are shown in Supplementary Figure S13B. **(B)** The percentage of infected cells (IE1-positive) with perinuclear accumulation (pn.) of PLEKHB2 was determined by epifluorescence microscopy and presented as mean ± SD of seven (Rab10) and four (EHBP1) independent experiments. The numbers in the bars indicate the total number of cells analyzed.

As indicated in our previous study (Lučin et al., 2020), the inner pre-AC contains multiple tubular compartments, and the expansion of the phosphoserine (PS)-rich domain, which can be characterized by the detection of the endogenous RE-resident PS-binding protein PLEKHB2 (Evectin-2) (Hasegawa et al., 2021), was also observed early in the infection. This domain is located in the EE/ERC pathway downstream of Rab10-PD at Rab11-ERC and likely represents tubular intermediates that migrate from the ERC to the Golgi (Uchida et al., 2011; Hasegawa et al., 2021). Therefore, to investigate whether Rab10 and EHBP1 are essential for pre-AC biogenesis, we monitored the expansion of PLEKHB2-PD after knockdown of Rab10 and EHBP1 using siRNA. As expected, knockdown of Rab10 and EHBP1 led to a significant reduction in Rab10 and EHBP1 accumulation, respectively, in the pre-AC (Figure 7A), consistent with the reduced expression of Rab10 (Supplementary Figure S13) and EHBP1 (Figure 5A). However, depletion of Rab10 and EHBP1 did not prevent the expansion of the PS-rich domain, as shown by the accumulation of PLEKHB2 in the pre-AC of Rab10-and EHBP1-depleted MCMV-infected cells (Figures 7A, B). These data indicate that Rab10-PD is not required for the expansion of PLEKHB2-PD, the downstream tubular domain of Rab11-ERC, suggesting that the recruitment of Rab10 to membranes of MCMV-infected cells is not required for the formation of the inner pre-AC.

## 4 Discussion

**FIGURE 8:**
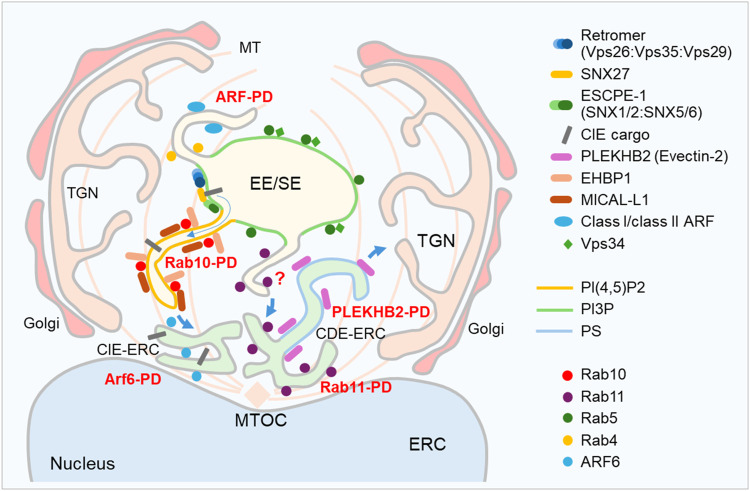
Schematic representation of the early events in the biogenesis of pre-AC after infection with MCMV. At 5–7 hpi, the Golgi is unlinked and displaced into a ring-like configuration (outer pre-AC) around expanded EE, RE/ERC and TGN, which form the inner pre-AC. Membrane recruitment and accumulation of host cell proteins associated with endosomal tubulation indicate the expansion of class I ARF-, class II ARF-, ARF6-, Rab10-, Rab11-and PLEKHB2-positive domains (Lučin et al., 2020; Pavišić et al., 2021). For simplicity, class I and class II ARFs are represented as one domain, although studies indicate different domains in endosomes. The formation of Rab10-PD requires the activity of Retromer:SNX27-ESCPE-1 complexes (Štimac et al., 2024), which are known to contribute to the retrieval of clathrin-independent endocytic (CIE) cargo, followed by EHBP1-dependent recruitment of Rab10 to PI(4,5)P-positive tubular membranes and prolonged retention of Rab10 at the membranes, leading to the accumulation of Rab10 in the inner pre-AC. Rab10 tubular membranes recruit MICAL-L1, which interacts with Rab10. The requirement of Retromer:SNX27:ESCPE1 and EHBP1 indicates expansion of EE-derived tubules, and Rab10 is not recruited by downstream Rabin8-dependent activation. The expansion of phosphatidylserine (PS)-rich domains of ERC that bind PLEKHB2 is independent of Rab10-PD formation, suggesting that the inner pre-AC contains multiple subsets of expanded tubular domains and that the CMV targets mechanism which is associated with termination of endosomal tubulation. Blue arrows indicate known trafficking routes. CIE-ERC and CDE-ERC denote subsets of ERC membranes known to maintain sequestration of CIE and CDE cargo. TGN, trans-Golgi network; ERC, endosomal recycling compartment; MTOC, microtubule organizing center; MT, microtubule.

This study provides evidence for the early expansion of Rab10-PD in the perinuclear region of CMV-infected cells, known as the inner pre-AC, which includes EEs, REs/ERC, TGN, and expanded membrane elements at the EE-RE/ERC-TGN interface (Lučin et al., 2018; Lučin et al., 2020). This evidence is based on confocal imaging of endogenous Rab10 and its interactors/effectors known to act at the EE-RE/ERC interface, long-term live cell imaging of EGFP-Rab10 using DHTM, and biochemical interactions of Rab10 with EHBP1 and MICAL-L1 determined using BioID. The Rab10-PD expansion requires EHBP1 and PI(4,5)P2, as depletion of EHBP1 by siRNA and saturation of PI(4,5)P2 with EGFP-PH-PLC-δ1 prevents perinuclear EHBP1 recruitment and Rab10 accumulation. These data, which include essential components of tubular recycling endosomes (Etoh and Fukuda, 2019; Farmer et al., 2021), together with our recent findings on the expansion of SNX27:Retromer:ESCPE-1-dependent membranes (Štimac et al., 2024), suggest an expansion of the tubular domain(s) at EEs in the pre-AC. The majority of Rab10-PD in pre-AC does not arise from the expansion of tubular domains downstream of Rab11, as Rabin8, a Rab11-dependent GEF that activates Rab10 at a Rab11 subset of the ERC (Homma and Fukuda, 2016), barely associates with Rab10 and its depletion has no effect on Rab10-PD expansion. As shown by the Rab10 depletion experiments, Rab10 itself is not required for the reorganization of the membrane system that leads to the establishment of the pre-AC, at least not for the part that includes expansion of other downstream tubular domains such as. PS-rich tubular domains that arise at the ERC. Thus, Rab10-PD is a subset of EE-derived tubular domains that are expanded in the E phase of infection and contribute to the formation of the inner part of the AC (Figure 8), which is fully developed in the L phase of infection.

In uninfected 3T3 fibroblasts, Rab10 is likely activated in a highly dynamic domain of EEs and downstream of Rab11-dependent recycling intermediates that form the Rab11 subset of ERC (Homma and Fukuda, 2016). The residence time of Rab10 at the EE domain and derived transport intermediates is quite short, as shown by imaging in living cells (Babbey et al., 2006; Schuck et al., 2007), which is hardly observable by immunofluorescence imaging and explains the weak Rab10 signal in 3T3 fibroblasts. The residence time of Rab10 is slightly longer at the ERC intermediates, which are concentrated in the pericentriolar region, as shown by the introduction of EGFP-Rab10. The residence time of Rab10 at the EE membranes is prolonged in HeLaM cells that constitutively generate TREs. In these cells, Rab10, MICAL-L1 and EHBP1 are essential components for the biogenesis and maintenance of EE-derived TREs that occur at membranes enriched in phosphoacetate and PI(4,5)P2 (Etoh and Fukuda, 2019; Farmer et al., 2021). In MCMV-infected cells, Rab10 accumulates in the pericentriolar area very early in infection (6-7 hpi in 3T3 fibroblasts) and Rab10-PD expands into the larger perinuclear region that concentrates EE membranes as the E phase of infection progresses from 6 to 16 hpi. These features of the faint fluorescent signal of endogenous Rab10 recruitment in uninfected cells and the bright fluorescent signal emanating from the pericentriolar region of the CMV-infected cell make the detection of Rab10 a useful biomarker for the reorganization of the membrane system and the establishment of the structure known as pre-AC/AC. Since the topology of the AC is similar in MCMV- and HCMV-infected cells, the analysis of pre-AC events in MCMV-infected cells is relevant for understanding the events in cells infected with HCMV and other BHVs.

A similar pattern of membrane recruitment to Rab10 is also observed for EHBP1, an essential host cell component required for Rab10 recruitment to membranes (Wang et al., 2016) and Rab10-PD expansion in MCMV-infected cells. Accordingly, most of the downstream Rab10 interactors/effectors examined in this study (AS160, TBC1D2, ACAP1, and ACAP2) showed similar behavior to Rab10, suggesting that the entire group is recruited to highly dynamic Rab10/EHBP1-dependent EEs and EE-derived intermediates. Rab10-PD development in MCMV-infected cells requires SNX27-initiated cargo retrieval machinery that assembles SNX27:Retromer:ESCPE-1 complexes at the EE membrane to sort CIE cargo and initiate endosomal tubulation (Štimac et al., 2024). This SNX27-dependent process also appears to be highly dynamic, and endogenous SNX27 shows similar properties to Rab10: a low retention time on the membranes of uninfected cells, generating a faint fluorescent signal, and a prolonged retention time in MCMV-infected cells, generating a bright fluorescent signal for membrane retention and expansion (Farmer et al., 2021; Štimac et al., 2024). The retention of membrane flux and the expansion of a highly dynamic EE domain that sorts CIE cargo is thus a biomarker for the earliest step of pre-AC biogenesis. Other EE domains associated with tubulation (Figure 8), such as domains organized by class I and class II ARFs (Pavišić et al., 2021), and ERC domains, such as domains characterized by Arf6, Rab11 and PLEKHB2 (Lučin et al., 2020; Pavišić et al., 2021), are also expanded within pre-AC in a similar pattern to Rab10 and may also serve as useful biomarkers for characterizing the earliest stages of membrane reorganization into AC (Figure 8). Class II ARFs, for example, are also barely endogenously recruited to membranes of uninfected cells and are highly recruited at 16 hpi but not at six hpi (Pavišić et al., 2021) and can therefore serve as a suitable marker to distinguish at least two steps in the biogenesis of pre-AC.

Although immunofluorescence studies showed an accumulation of Rab10 effectors in MCMV-infected cells, BioID did not detect any significant interaction with Rab10. The interaction network that regulates membrane flux, including the Rab10 network, is quite complex and still poorly understood. To understand the principles that expand tubular domains within the pre-AC and drive membrane reorganization, including factors encoded by the virus, it would be important to identify the downstream mechanisms affected by infection. For example, BioID does not detect intense interaction of AS160, a known GAP protein required for Rab10 inactivation (Homma et al., 2021), but AS160 is highly recruited to pre-AC membranes. Therefore, it is reasonable to speculate that the inability to turn off Rab10 could lead to the expansion of Rab10-PD. However, further studies are needed, including widening the list of Rab10 interactors, as a much broader list of Rab10 interactors was identified in a recent study using BioID and proteomic characterization (Zhu et al., 2024).

The proposed sequence for the formation of Rab10-PD is shown in Figure 8. Expansion of Rab10-PD in the pre-AC requires activation of SNX27:Retromer:ESCPE-1 complexes at EE (Štimac et al., 2024), which are known to retrieve CIE cargo and initiate tubulation of EE membranes (Cullen and Steinberg, 2018). SNX5, a component of ESCPE-1, may provide a platform for further progression of tubulation by associating with PIPKIγi5, an endosomal variant of PIP5K that generates PI(4,5)P2 (Tan et al., 2015). EHBP1 binds directly to PI(4,5)P2 via its N-terminal C2-like domain, connects the endosome to actin via its central CH domain, and specifically recruits Rab10-GTP via its C-terminal CC domain (Wang et al., 2016). Rab10 itself can link motor proteins to microtubules, which is required for membrane elongation and tubule growth (Etoh and Fukuda, 2019). Tubulation is also supported by the recruitment of MICAL-L1, another important component of TREs that interacts with Rab10 (Fukuda et al., 2008) and whose recruitment is likely facilitated by Rab10 (Farmer et al., 2021). Prolonged retention of Rab10 on membranes suggests a defect in termination of the tubulation reaction, which could be targeted by CMV-encoded functions expressed in the E phase of infection. Therefore, the increasing interaction of MICAL-L1 with Rab10 in pre-AC points to a specific target for further studies to explain the extensive tubulation within pre-AC, including the expansion of Rab10-PD. For example, MICAL-L1 may fail in assembling complexes with Pacsin2 and EHD1, which may be important for termination of tubulation by for tubule fission (Farmer et al., 2021).

### 4.1 Functional consequences of membrane tubulation in the pre-AC

The reorganization of the membrane system in the E phase of infection into the pre-AC, leads to a reorganization of the functional trafficking networks (Mosher et al., 2022), which differs significantly from those of uninfected cells (Beltran et al., 2016; Zeltzer et al., 2018; Cook and Cristea, 2019). Many aspects of these processes are not yet well understood, mainly due to their complexity and insufficient knowledge of these processes in uninfected cells (Mosher et al., 2022; Turner and Mathias, 2022). These complex reorganizations may have several known and important functional consequences for the biology and pathogenesis of CMV infection, such as (i) alteration of the cell surface proteome of a large number of cell proteins, such as proteins involved in apoptosis, cell adhesion, immune response, metabolism, transport and signaling (Gudleski-O’Regan et al., 2012; Weekes et al., 2014; Beltran et al., 2016); (ii) concentration of viral glycoproteins, including viral envelope proteins; (iii) control of intracellular signaling by reorganizing the transport pathways of signaling receptors by retaining some receptors in endosomes or redirecting some receptors for degradation, such as EGFR in fibroblasts infected with HCMV (Rak et al., 2018); (iv) establishment of a signaling platform for viral gene expression (Štimac et al., 2024); (v) immune evasion by delayed transit through EEs, retention in EEs and inhibition of endosomal recycling, as observed in HCMV- (Boeck and Spencer, 2017; Zeltzer et al., 2018) and MCMV- (Tomaš et al., 2010; Karleuša et al., 2018) infected cells, which subverts recognition by cytotoxic T lymphocytes (Lučin et al., 2015) and NK cell responses (De Pelsmaeker et al., 2018); (vi) inhibition of endosomal acidification and modulation (inhibition) of autophagic flux and degradation (Lee et al., 2021; Li et al., 2024) or undermining of efficient MHC class II presentation and efficient signaling through TLRs (Li et al., 2024); and (vii) virion assembly and egress.

### 4.2 How Rab10-positive tubular membranes can contribute to the virion assembly?

Expanded Rab10-PD into tubular extensions may serve as a source of membranes for secondary envelopment. The retarded membrane flux in these tubular domains may serve to retain important components required for the formation of a favorable environment for the secondary envelopment. This is mainly related to the concentration of envelope glycoproteins on the membranes of the endosomal system used for envelopment of the virions, as well as the concentration of other viral glycoproteins required for the formation of the biomolecular condensate of the tegument matrix on the membranes used for envelopment. However, the pathways of viral glycoprotein sorting in the distal part of the EE route are largely unknown. In our recent study (Štimac et al., 2024), we reported that the expansion of Rab10-PD depends on the activity of SNX27:Retromer:ESCPE-1 complexes, which are mainly associated with the retrieval of CIE cargo (Cullen and Steinberg, 2018). Thus, this pathway may serve to sort some viral glycoproteins into Rab10-positive tubular extensions. Although not all viral glycoproteins follow the CIE trafficking pathway and are retained in the corresponding tubular intermediates, there are other pathways that can be utilized for the retention of viral glycoproteins, such as the SNX3-dependent cargo retrieval pathway. Furthermore, in addition to these two predominant retrieval mechanisms at the EEs, there are other sorting mechanisms that may serve to deliver glycoproteins to other tubular intermediates that are enriched in the inner AC. To address these questions, it would therefore be important to establish experimental models and map the endosomal transport pathways for all viral glycoproteins. This would help to clarify the mechanism used for secondary envelopment. EM studies (Maninger et al., 2011; Schauflinger et al., 2013; Taisne et al., 2019; Momtaz et al., 2021; Flomm et al., 2022) suggest the wrapping-based mechanism. The tubular membranes derived from the RE system able to wrap around a large condensate of tegumented single or multiple capsids, as described for phagophore development. A recent study by Puri et al. (Puri et al., 2023) showed that multiple origin foci on REs are used to develop autophagosomes by forming finger- or octopus-like projections that wrap around the autophagic material and close into autophagosomes by ESCRT- and dynamin-dependent mechanisms. The octopus-like model of the envelopment suggests that tubular projections of different origins are used to develop the wrapping sac, which may explain the heterogeneity of envelope composition observed in CMV studies. EM observations (Maninger et al., 2011; Schauflinger et al., 2013; Taisne et al., 2019; Momtaz et al., 2021; Flomm et al., 2022), functional studies (Archer et al., 2017; Bergner et al., 2024), the need for ESCRT-III (Tandon et al., 2009; Streck et al., 2018) and dynamin (Hasan et al., 2018; Štimac et al., 2021) for maturation and release of CMV virions, and the host cell signatures within virions (Mahmutefendić Lučin et al., 2022) identified in proteomic (Birzer et al., 2020; Couté et al., 2020; Turner et al., 2020; Flomm et al., 2022) and lipidomic (Liu et al., 2011) studies of purified virions, argue in favor of a wrapping-based mechanism. Rab10-positive expanded intermediates may serve as a source of membranes for wrapping around virions by generating downstream tubular membranes, such as Rab15 membranes. The role of the Rab10-EHBP1-EHD2 complex in the development of wrapping membranes has been demonstrated in the assembly of lipophagic machinery in hepatocytes (Li et al., 2016), where Rab10 promotes membrane expansion and assembly of the machinery required for the extensive mechanochemical work to deform, stretch and expand an autophagic membrane along the lipid droplet surface.

### 4.3 Conclusion

The present work indicates that pre-AC biogenesis is complex and likely involves multiple parallel processes, including the expansion of the Rab10-PD. The expanded Rab10-PD in CMV-infected cells represent EE-derived tubular endosomal membranes based on EHBP1-dependent Rab10 recruitment to the CIE cargo sorting tract on EE endosomes. Rab10-associated tubulation occurs early in infection may serve as a biomarker for the earliest changes in the establishment of pre-AC that can be monitored by immunofluorescence. Our experiments, particularly our silencing experiments, show that Rab10 and EHBP1 do not contribute significantly to the later stages of inner pre-AC biogenesis or to the expansion of downstream tubular domains. A more comprehensive understanding of the significance of tubular domain expansion remains to be determined in further studies. Indeed, the restructuring of the membrane system in CMV-infected cells is extensive and functional interpretation and study of these changes based on mere repositioning of normal cellular processes is difficult, as there is little homology with normal cellular processes (Moorman et al., 2010; Procter et al., 2018). To understand the new functional order in the membrane system created by infection, it would be important to investigate how the virus utilizes host resources to build the pre-AC, including the establishment of the viral factory at later stages of infection. This requires an understanding of the composition and organization of the membrane domains within the pre-AC, and the present study contributes to this. As expected, this knowledge and the expansion of some cellular functions may facilitate further progress in understanding these processes in normal, uninfected cells, as the virus is the best cell biologist.

## Data Availability

The datasets presented in this study can be found in online repositories. The names of the repository/repositories and accession number(s) can be found in the article/Supplementary Material.
