# Supplementary material for: Rab10-associated tubulation as an early marker for biogenesis of the assembly compartment in cytomegalovirus-infected cells
Source: Front Cell Dev Biol. 2025 Jan 10;12:1517236. doi: 10.3389/fcell.2024.1517236 (PMC11760598; doi:10.3389/fcell.2024.1517236)
Supplement: Supplementary file 3 [file DataSheet1.pdf]

Supplementary Material

1 Supplementary Data

2 Supplementary material and methods

2.1 Design of Rab10-BioID2-HA construct and production of pGenLenti Rab10-BioID2-HA lentiviral plasmids

To design Rab10-BioID2-HA fusion construct, sequence of murine Rab10 was retrieved from Ensembl genome database, ensesmbl.org). Humanized BioID2-HA sequence was achieved from MCS-BioID2-HA plasmid (a gift from Kyle Roux (Addgene plasmid # 74224 ; http://n2t.net/addgene:74224 ; RRID:Addgene_74224)), and was codon-optimized for expression in murine cells with GenSmart™ Codon Optimization tool (Patent Number: WO2020024917A1). Finally, 13x(GGGGS) linker (Kim et al, MBoC, 2016) was inserted between Rab10 and BioID2-HA sequences. The final construct :
(atggcgaagaagacgtacgacctgcttttcaagctgctcctgatcggggactcgggagtgggcaagacctgcgtcctttttcgtttttcggacgatgccttcaata
ccacctttatttccaccataggaatagactttaagatcaaaacagtggaactacaaggaaagaagatcaagctacagatatgggacacagcaggccaggagcg
atttcacaccatcacaacctcctactacagaggagcaatgggcatcatgctagtgtatgacatcaccaacggtaaaagctttgagaacatcagcaagtggcttaga
aacatagatgagcatgccaatgaagatgtggaaagaatgttactagggaacaagtgtgacatggacgacaagagagttgtaccgaaaggcaaaggagaaca
gattgcaagggagcatggtattaggtttttgagactagtgcaaaagcaaatataaacatcgaaaaggcgttcctcacattagctgaagacatcctccgaaagacc
cctgtaaaagaacccaacagtgaaaacgtagatatcagcagtggaggaggcgtgacgggctggaagagcaagtgctgcggtggaggcgggtctggaggcg
gggtagtggcgggggtggaagcgggggtggaggcgggtcgggtggcggaggtagcggaggcggtggaagtggaggcggtggaagtggtggagga
ggttctggtggcggaggtagcggtggcggaggtagcggaggcggtggaagtggtggcggaggtagcggaggcggtggatcgTTTAAGAACTTG
ATCTGGCTAAAAGAAGTCGACTCCACACAGGAGAGGCTTAAAGAGTGGAATGTCTCCTATGGCACCG
CTCTTGTAGCTGATAGGCAAACCAAGGGCCGGGGTGGCCTTGGAAGAAAATGGCTGAGCCAAGAAG
GAGGTCTTTATTTCAGCTTCCTGCTGAATCCCAAGGAATTTGAGAATCTTCTGCAGCTGCCTTTAGTGT
TAGGTCTATCAGTTTCTGAAGCCCTGGAGGAGATCACAGAGATTCCCTTCTCCTTAAAGTGGCCGAAT
GATGTGTACTTCCAGGAAAAAAAAGTCAGTGGGGTGCTCTGCGAGCTGAGCAAAGACAAGCTCATAG
TAGGAATTGGAATCAACGTGAACCAGCGCGAAATACCAGAGGAAATTAAGGATCGCGCGACGACTC
TGTATGAGATAACTGGAAAAGACTGGGACAGAAAGGAAGTTCTTCTGAAGGTGTTGAAGCGGATCTC
GGAGAACCTCAAGAAGTTCAAGGAGAAATCCTTTAAAGAGTTCAAAGGGAAGATTGAAAGCAAGAT
GCTGTACCTGGGGGAAGAGGTGAAGCTGTTGGGTGAGGGCAAGATCACCGGGAAACTGGTGGGCCTC
AGTGAGAAAGGAGGCGCACTGATTCTCACAGAAGAAGGCATCAAGGAAATCCTCTCAGGGGAGTTTT
CTTTGCGTCGATCTTACCCATATGATGTTCCTGACTACGCCTGA)

3 Supplementary Figures and Tables

3.1 Supplementary Figures

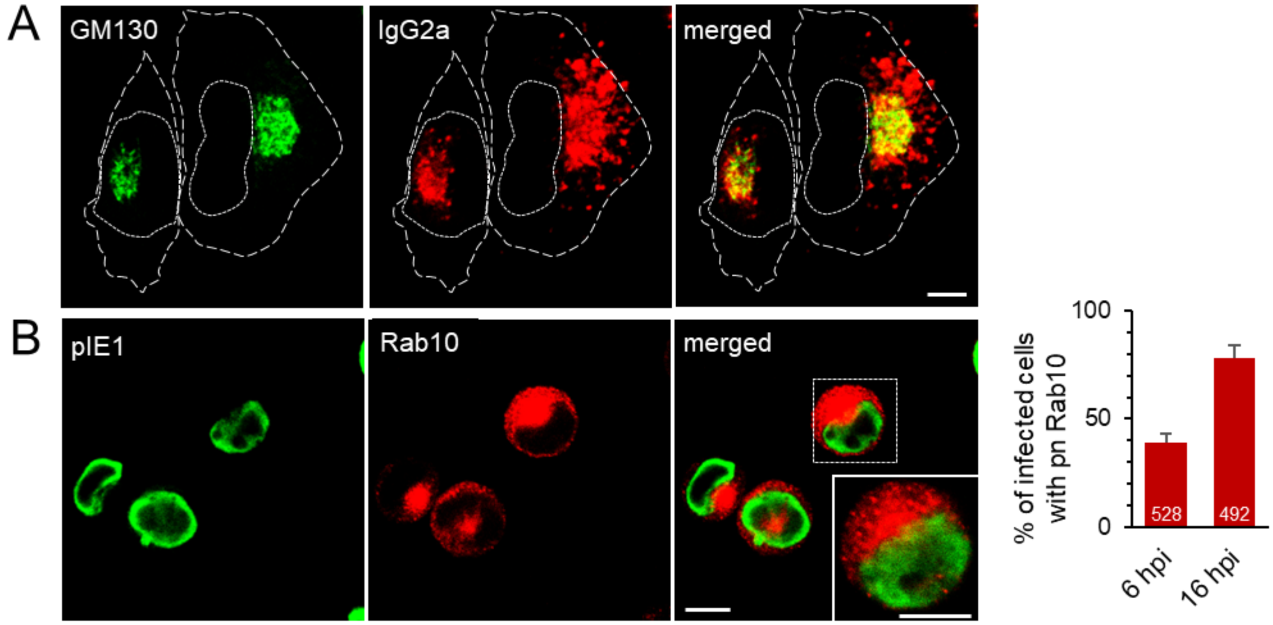

Figure S1. Expansion of Rab10-PD in cells infected with MCMV expressing m138 (*related to Figure 1A*). Balb 3T3 cells were infected with w.t. MCMV (MOI of 10), fixed 6 and 16 hours post infection (hpi), permeabilized and double stained for confocal analysis. (**A**) Infected cells (16 hpi) stained with Abs against the Golgi marker GM130 (green) in combination with IgG2a mouse mAb W6/32 (red). The antibody W6/32 specifically recognizes the heavy chain of the human MHC class I proteins and does not react with the mouse MHC class I proteins. This antibody does not react in immunofluorescence with any component of Balb 3T3 cells and is recognized by m138 protein (FcR) in MCMV-infected cells. Cell borders are indicated by fine dashed lines and nuclei by fine dotted lines. (**B**) Infected cells (16 hpi) stained with Abs against Rab10 (red) in combination with Abs against pIE1 (green). The percentage of cells with perinuclear (pn) accumulation of Rab10 in MCMV-infected (pIE1-positive) cells is shown as mean ± SD of three independent experiments. The numbers in the bars indicate the total number of cells analyzed. Bars, 10 μm.

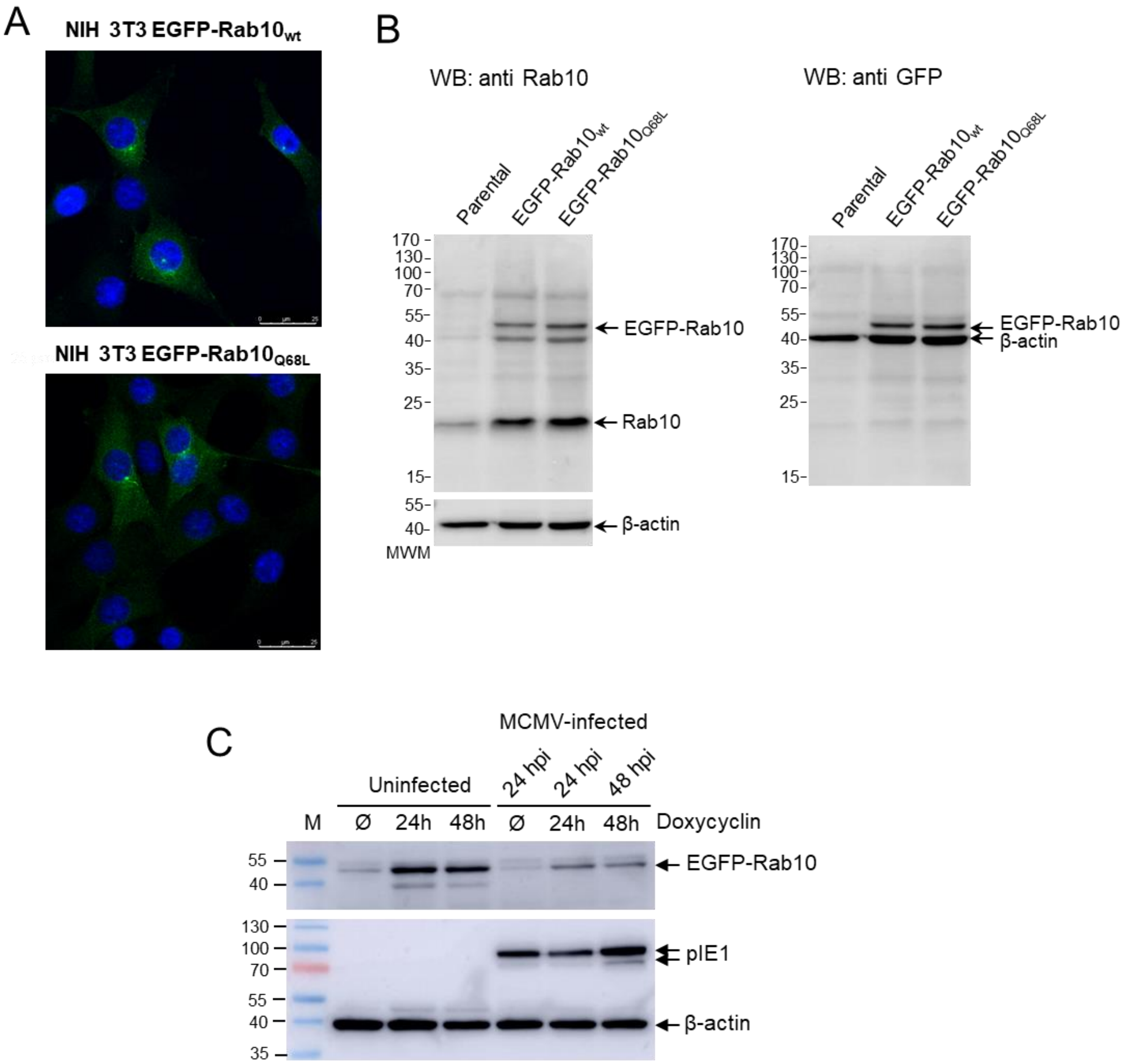

Figure S2. NIH 3T3 cell lines with inducible expression of EGFP-Rab10 constructs (*related to Figure 1B*). NIH 3T3 EGFP-Rab10$_{wt}$ and NIH 3T3 EGFP-Rab10$_{Q68L}$ cells were treated with doxycycline (DOX; 2 µg/ml) and analyzed after 24 hours for (**A**) EGFP-Rab10 fluorescence by confocal imaging and (**B**) Rab10 expression by Western blot using anti-GFP and anti-Rab10. (**C**) EGFP-Rab10$_{wt}$ does not over accumulate in uninfected and MCMV-infected NIH 3T3 EGFP-Rab10 cells after induction with DOX. NIH 3T3 EGFP-Rab10$_{wt}$ cells were treated with DOX or infected with Δm138-MCMV (MOI of 10) and treated with DOX after infection. Uninfected cells were collected for Western blot analysis before (Ø) or 24 and 48 hours after treatment with DOX. Untreated MCMV-infected cells (Ø) were collected for Western blot after 24 hours, and DOX-treated MCMV-infected cells were collected at 24 hpi and 48 hpi. The cell samples were lysed in RIPA buffer, separated by SDS-PAGE at 13% gel, blotted onto the PVDF membrane and probed with rabbit antibodies against GFP (1:2000). Expression of pIE1 and β-actin in each sample served as infection and loading controls, respectively, and was performed on the same membranes. Signals were quantified using ImageJ, normalized to actin signal, and expressed as arbitrary units relative to the strongest signal.

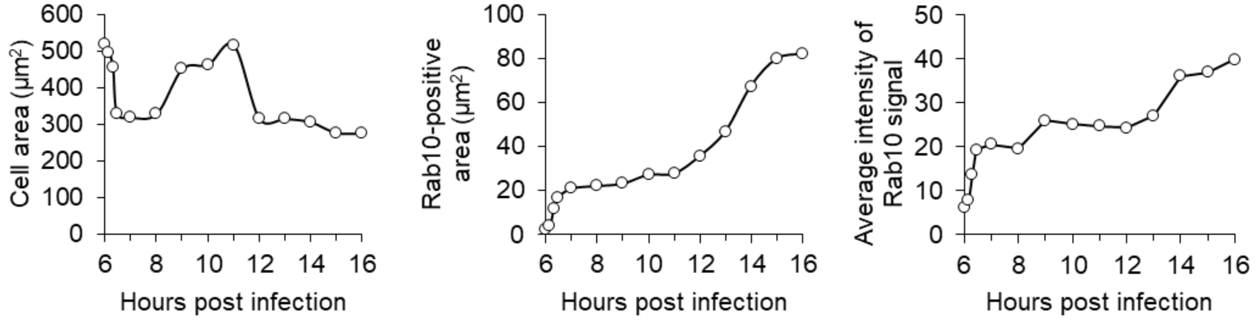

Figure S3. Cell area, Rab10-positive area, and mean fluorescence intensity of the Rab10-positive area of the cell during time-lapse imaging (6-16 hpi) of the cell shown in Figure 1C. Expression of EGFP-Rab10 in the NIH 3T3 EGFP-Rab10 cell line was induced by doxycycline (2 μg/ml), and after 24 hours, cells were infected with Δm138-MCMV (MOI of 10). Cells were imaged continuously from 6 hpi with DHTM at intervals of 2.5 min for refractive index (RI) and 5 min for fluorescence signal. The frames at the indicated times of 6-16 hpi were extracted as raw images from STEVE (TIFF) and processed with ImageJ. The cell area was calculated on the RI image through the focal plane and the Rab10-positive area on the fluorescence image using the freehand tool and the region of interest (ROI) manager. The average fluorescence intensity of the Rab10-positive area was calculated on the fluorescence image using the Region of Interest (ROI) Manager tool.

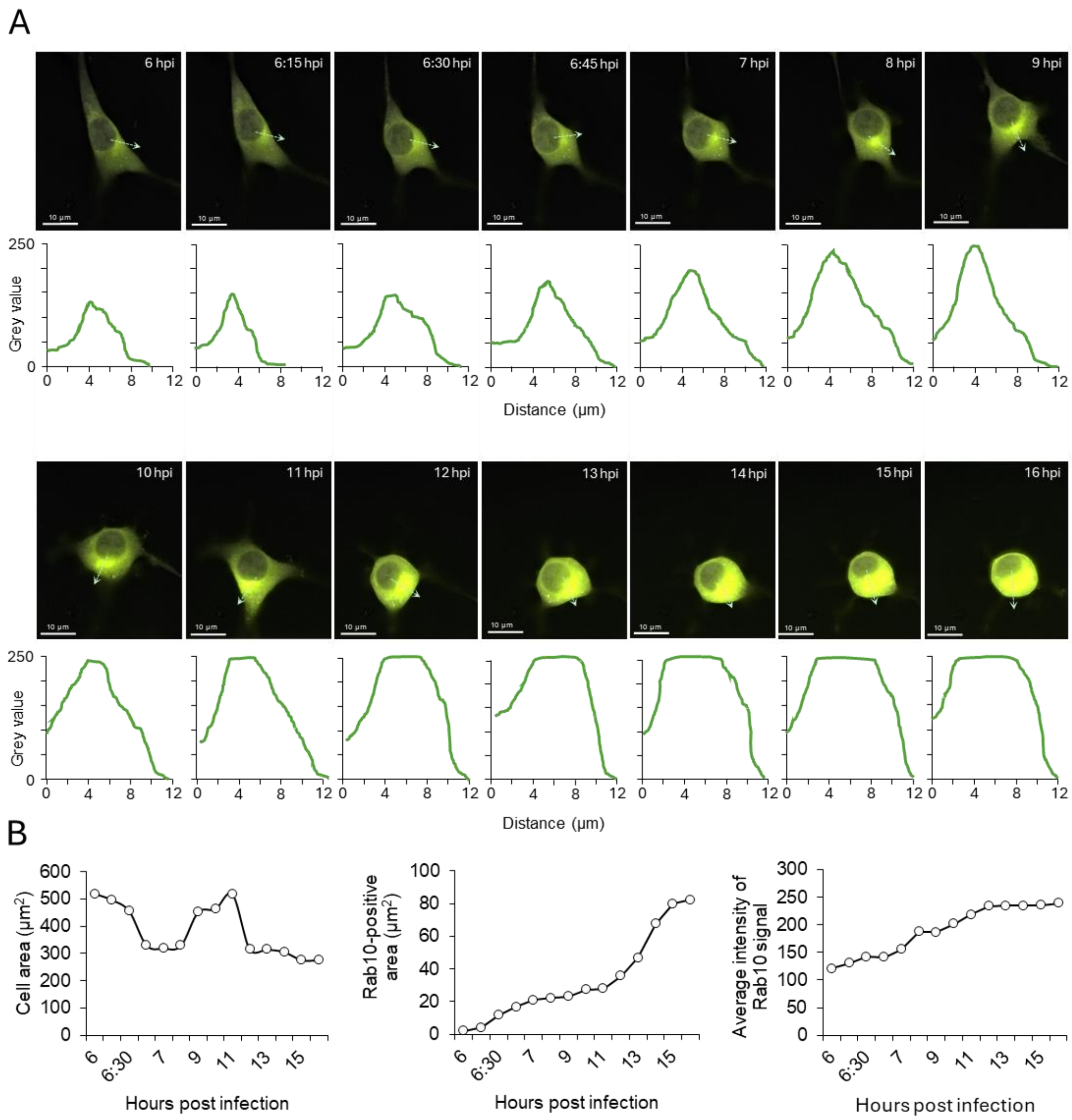

Figure S4. Long-term high-frequency live cell imaging of MCMV-infected cells using DHTM with attached epifluorescence module (*related to Figure 1C*). (**A**) Expression of EGFP-Rab10 in the NIH 3T3 EGFP-Rab10 cell line was induced by doxycycline (2 µg/ml), and after 24 hours, cells were infected with Δm138-MCMV (MOI of 10). Cells were imaged continuously from 6 hpi with DHTM at intervals of 2.5 min for refractive index (RI) and 5 min for fluorescence signal. The overlaid images show RI and fluorescence signal at one-hour intervals during the E phase of infection (6-17 hpi). The RI and fluorescence recording, and the overlayed video are shown in Video S4-S6. Fluorescence intensity profiles along light green dashed arrow lines are shown below the images, and the cell area, Rab10-positive area, and mean fluorescence intensity of the Rab10-positive area are shown in (**B**). Frames at the indicated time points of 6-16 hpi were extracted as raw images from STEVE (TIFF) and processed with ImageJ. The cell area was calculated on the RI image through the focal plane and the Rab10-positive area on the fluorescence image using the hand-free tool and the region of interest (ROI) manager. The average fluorescence intensity of the Rab10-positive area was calculated on the fluorescence image using the Region of Interest (ROI) Manager tool.

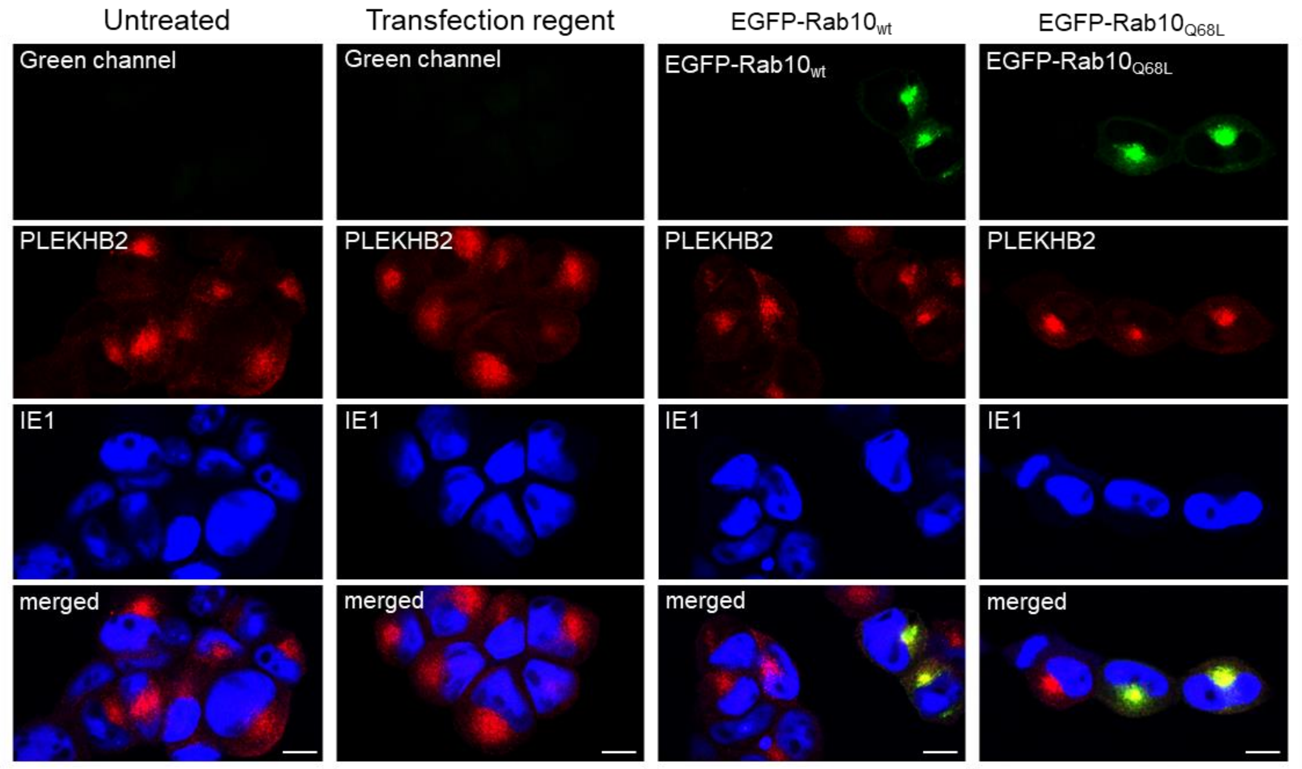

Figure S5. Accumulation of wild-type and GTP-locked Rab10 in the pre-AC (*related to Figure 1*). Untreated, transfection reagent-treated, EGFP-Rab10$_{wt}$- and EGFP-Rab10$_{Q68L}$-transfected Balb 3T3 cells were infected with Δm138-MCMV (MOI of 10) and 16 hours after infection stained with antibodies against PLEKHB2 (red) and pIE1 (blue). Transfected cells accumulated EGFP-Rab10$_{wt}$- and EGFP-Rab10$_{Q68L}$ (green fluorescence), while non-transfected cells accumulated endogenous Rab10 in the perinuclear region along with the accumulation of PLEKHB2, representing pre-AC. Bars, 10 μm.

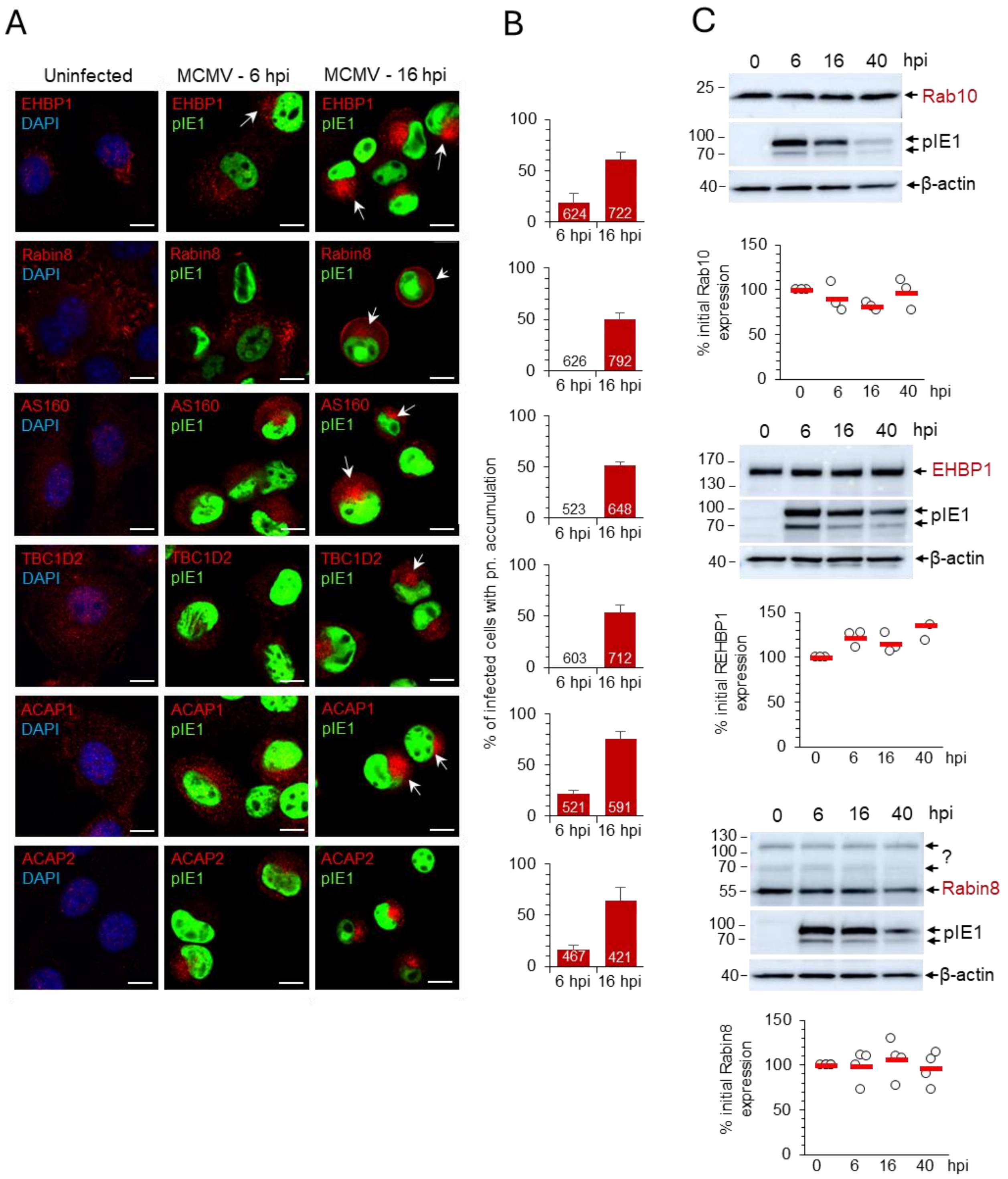

Figure S6. Perinuclear accumulation of EHBP1, Rabin8 and Rab10 effectors in the pre-AC of MCMV-infected Balb 3T3 cells (*related to Figure 2*). (**A**) Immunofluorescence analysis of EHBP1, Rabin8 and Rab10 effectors. Balb 3T3 cells were infected with Δm138-MCMV (MOI 10) or left uninfected, fixed at 6 and 16 hpi, permeabilized and stained with Abs against EHBP1, Rabin8, AS160, TBC1D2, ACAP1 or ACAP2 (red) in combination with Abs against pIE1 in infected cells to control infection (green) or with DAPI (blue) in uninfected cells to stain the nuclei. Shown are the merged confocal images through the focal plane of a representative experiment. The arrows indicate perinuclear accumulation in the

pre-AC. Bars, 10 µm. (**B**) Percentage of cells with perinuclear (pn) accumulation in MCMV-infected (IE1-positive) cells, shown as mean ± SD from three independent experiments. The numbers in the bars indicate the total number of cells analyzed. (**C**) Western blot analysis of Rab10, EHBP1 and Rabin8 expression during the E (6 and 16 hpi) and L (40 hpi) phase of infection. Expression of pIE1 and β-actin in each sample served as infection and loading controls, respectively, and was performed on the same membranes. Signals were quantified using ImageJ and expressed as a percentage of the initial expression. Shown are the individual results (empty circles) and the average (red bars) of three (Rab10 and EHBP1) and four (Rabin8) experiments.

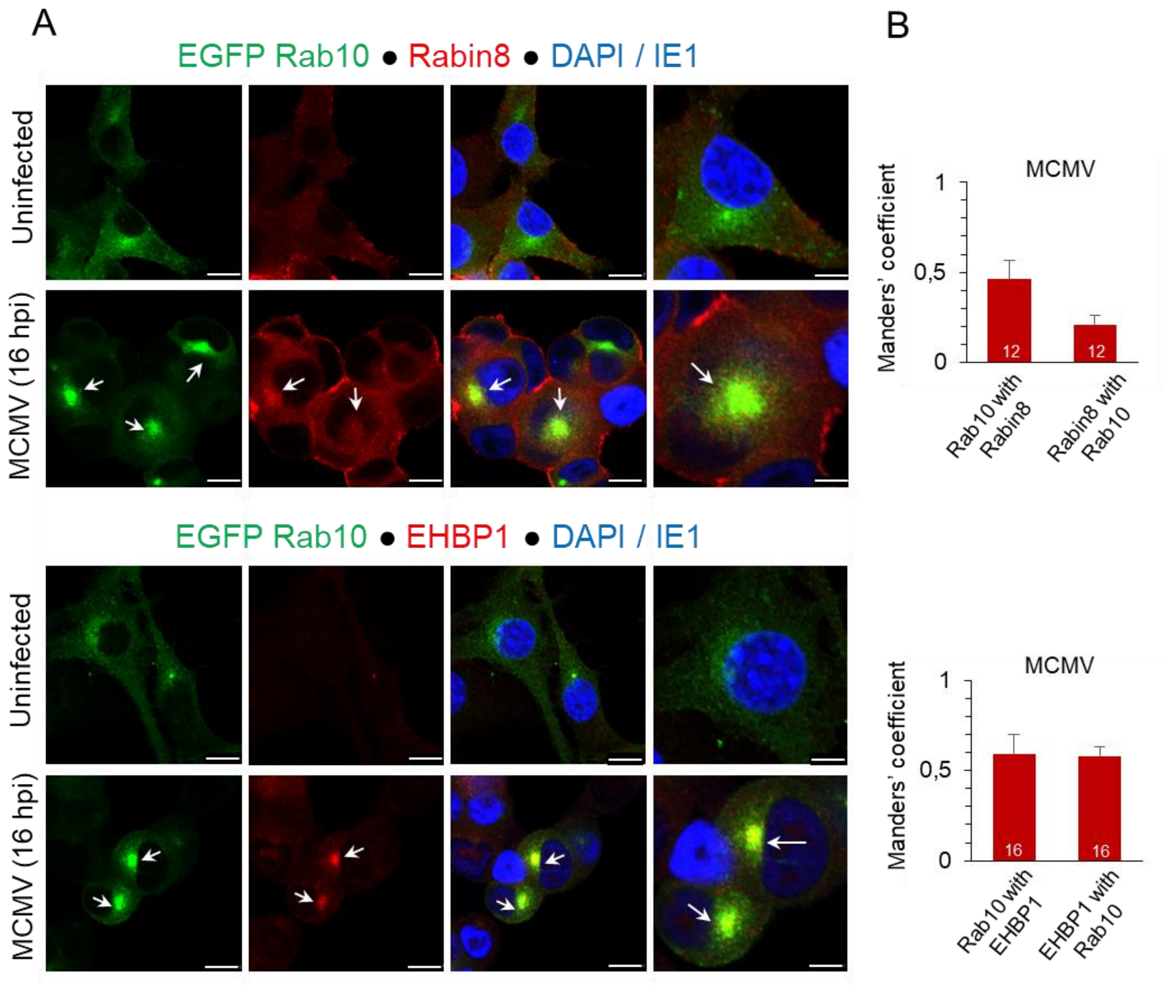

Figure S7. Colocalization of EGFP-Rab10 with Rabin8 or EHBP1 in uninfected and MCMV-infected cells. (**A**) NIH 3T3 EGFP-Rab10 cells were uninfected or infected with Δm138-MCMV (MOI of 10), fixed 16 hours later, permeabilized and stained with Abs against EHBP1 or Rabin8 (red) in combination with Abs against pIE1 (blue) in infected cells to control infection or DAPI (blue) in uninfected cells to stain nuclei. Shown are the merged confocal images through the focal plane of a representative experiment. The arrows indicate perinuclear pre-AC. Bars, 10 µm (**B)** Mander's coefficients (M1 and M2) were calculated on the entire image stacks to quantify colocalization. Data represents mean ± SD from two independent experiments. The numbers in the bars indicate the total number of cells analyzed.

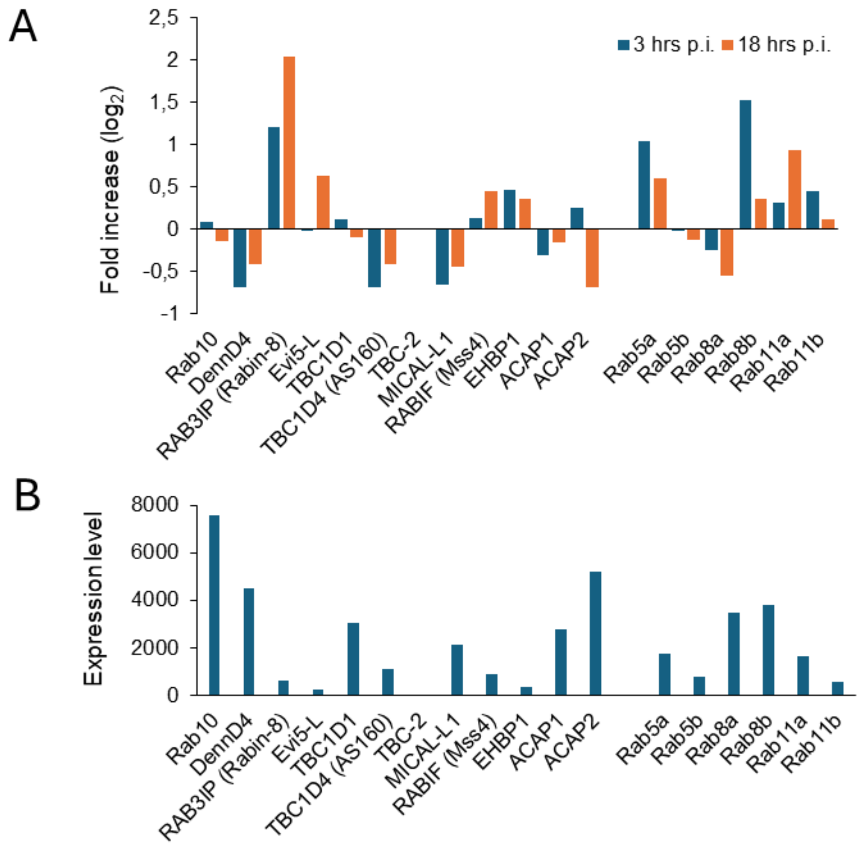

Figure S8. Effect of MCMV infection on the expression of the host cell genes encoding members of the Rab10 interaction network that control membrane flux at the EE-RE/ERC interface *(related to Figure 2)*. (**A**) The data represent the fold change (log_2) of gene expression at the beginning (3 hpi) and the end (18 hpi) of the early phase of MCMV infection relative to the mock-infected cells. (**B**) Absolute counts related to gene expression.

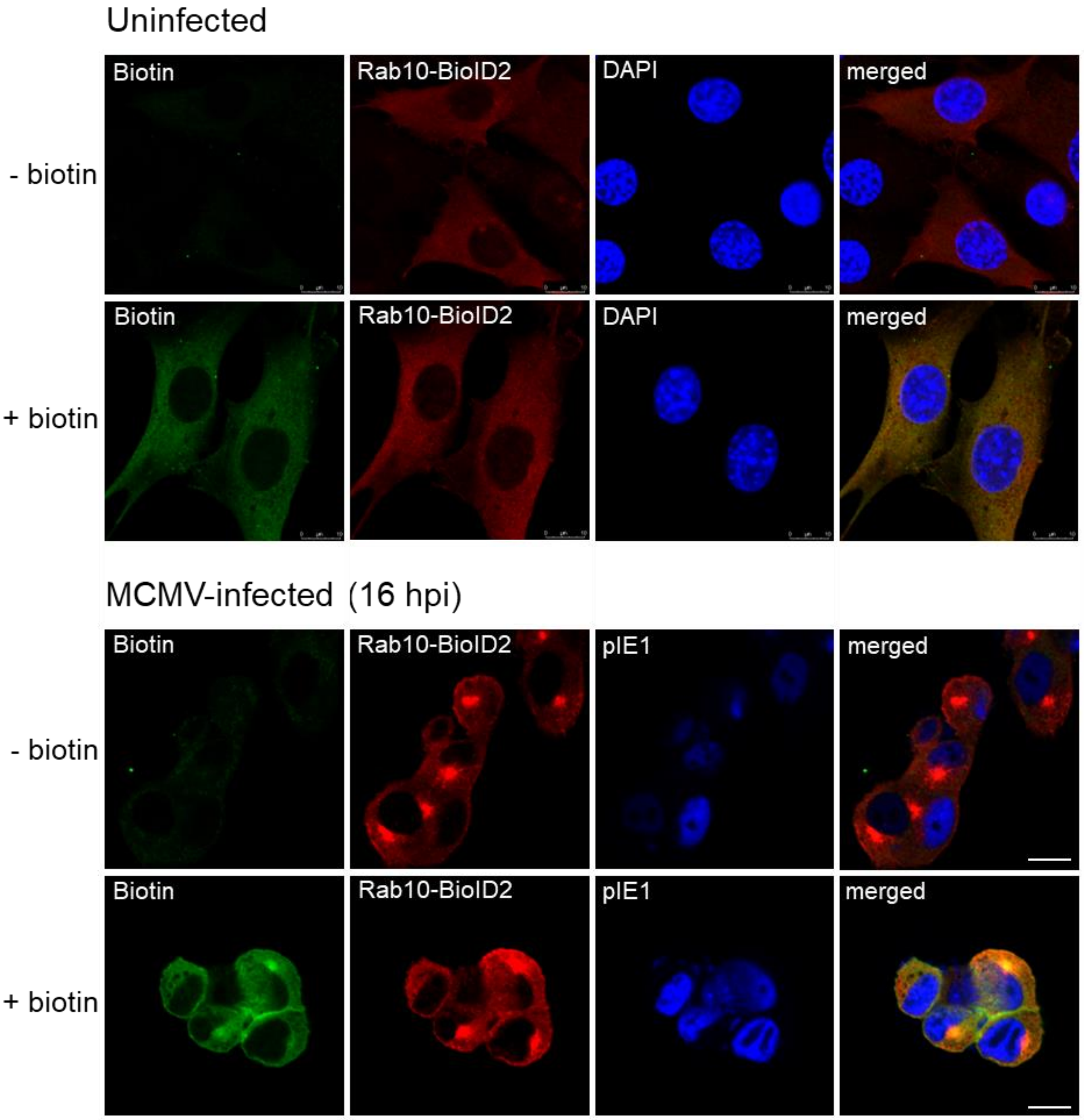

Figure S9. Biotinylation reaction in uninfected and MCMV-infected NIH 3T3 Rab10-BioID2-HA cell line (*related to Figure 3E*). Immunofluorescence analysis of uninfected and MCMV-infected (Δm138-MCMV at an MOI of 10 for 16 hours) cells treated with biotin (50 µM) for 16 hours or left untreated, fixed, permeabilized and stained with AF^{488}-conjugated streptavidin (SA) for visualization of biotinylated proteins, anti-HA for visualization of Rab10-BioID2-HA and anti-IE1 for visualization of pIE1, followed by AF^{555}- or AF^{680}-conjugated non-cross-reactive secondary antibodies. Bars, 10 µm.

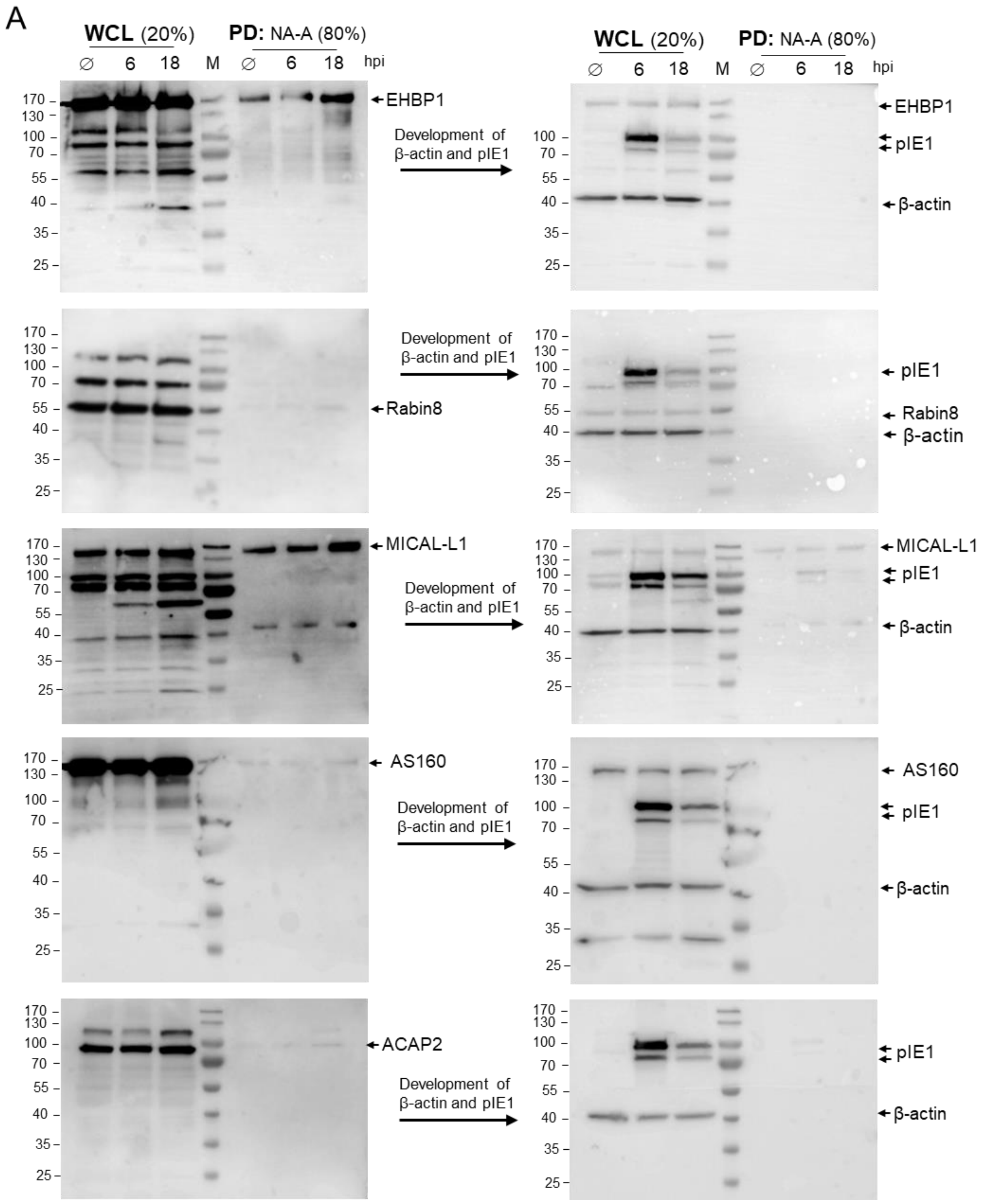
A
WCL (20%)
PD: NA-A (80%)
∅ 6 18 M ∅ 6 18 hpi
EHBP1
Development of β-actin and pIE1
pIE1
β-actin
Rabin8
MICAL-L1
AS160
ACAP2
170
130
100
70
55
40
35
25

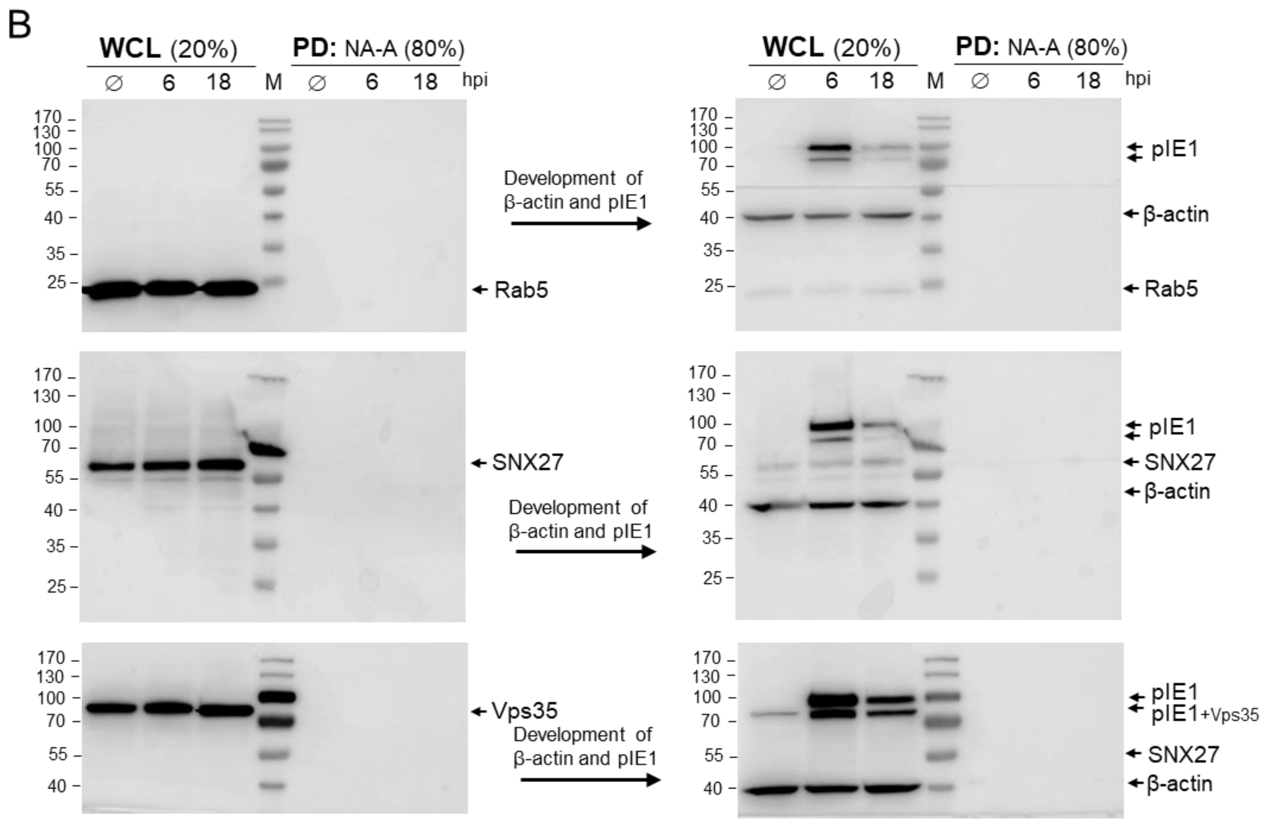

Figure S10. Original raw blots and unprocessed ECL images of representative Western blots from the proximity-dependent biotin identification (BioID) experiment shown in Figure 4 of the manuscript. NIH 3T3 Rab10-BioID2-HA cells were exposed to biotin and left uninfected for 18 hours (Ø) or infected with Δm138-MCMV (MOI of 10) at one time point or 12 hours post-biotin and incubated in biotin for a total of 18 hours to reach 18 and 6 hpi, respectively. Cell samples were lysed and 20% of the sample was used for Western analysis as whole cell lysate (WCL) and 80% of the sample was used for pull-down (PD) with NeutraAvidin-Agarose (NA-A) and subsequent Western blot analysis. The membranes with WCL and NA-A pull-down (PD) samples were stained with (**A**) antibodies against Rab10 interactors: (EHBP1, Rabin8, MICAL-L1, AS160 and ACAP2) and (**B**) non-interacting cellular proteins Rab5, SNX27 and Vps35. The original blots after staining with antibodies against host cell proteins are shown on the left, while the same membranes after subsequent staining for pIE1 and β-actin are shown on the right. The IE1 protein is shown as a phosphoprotein of 89 kDa (pp89) and in the pp78 form. The molecular weight markers used in these experiments are PageRuler Prestained Protein Ladder (Thermo Scientific, product: 26616, LOT: 00515508). A PVDF membrane was used for blotting (Merck Millipore, size: 0.45um, Immobilon-P Transfer Membranes CAT: IPVH00010, LOT: R6PA1239C). M, marker line; Ø, noninfected; 6 and 16, time post infection; hpi, hours post infection.

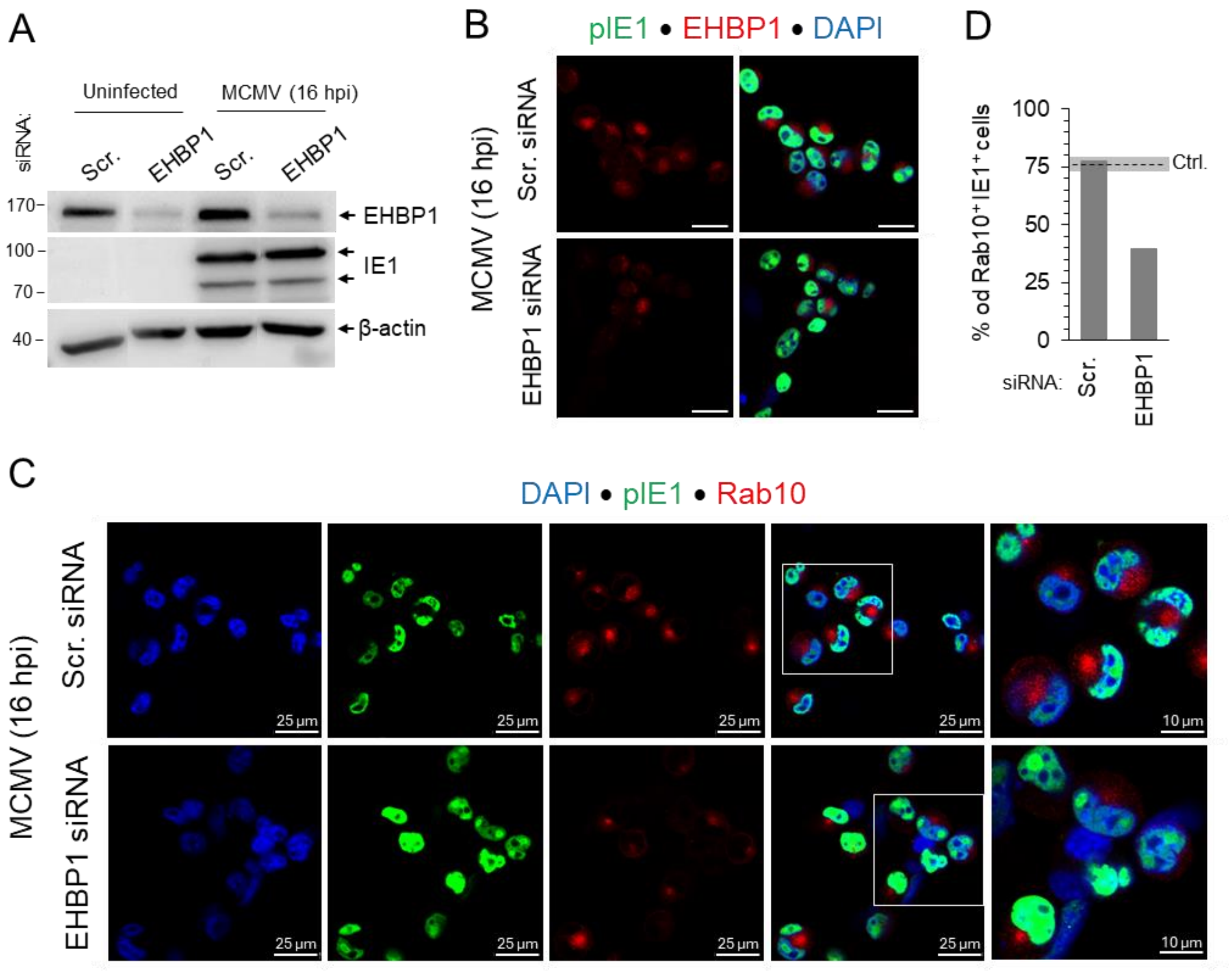

Figure S11. Depletion of EHBP1 prevents perinuclear expansion of Rab10-PD in the pre-AC of Balb 3T3 cells. Balb 3T3 fibroblasts were transfected with siRNA to EHBP1 (100 nM) or with “scrambled” siRNA (Scr.; 80 nM; Qiagen, 1022076). After 48 hours, cells were infected with Δm138-MCMV (MOI of 10) for 16 hrs or left uninfected. (**A**) Western blot and immunofluorescence analysis of EHBP1 expression in Scr. siRNA- and EHBP siRNA-treated cells at 16 hours post-infection. EHBP1 was determined by Western blot analysis on the same membrane together with pIE1, as a control for infection, and β-actin, as a loading control. (**B**) Immunofluorescence detection was performed by confocal imaging of endogenous EHBP1 staining (red) together with visualization of infection by pIE1 staining (green) and cell nuclei by DAPI staining. Shown are the confocal images through the focal plane. (**C**) Immunofluorescence detection of endogenous Rab10 (red) in Scr. siRNA- and EHBP siRNA-treated cells together with pIE1 (green) and DAPI. Shown are the confocal images through the focal plane. (**D**) Percentage of MCMV-infected (pIE1-positive) cells with perinuclear accumulation of Rab10 in Scr. siRNA- and EHBP siRNA-treated cells 16 hpi. Ctrl., control level in non-transfected cells (mean ± SD).

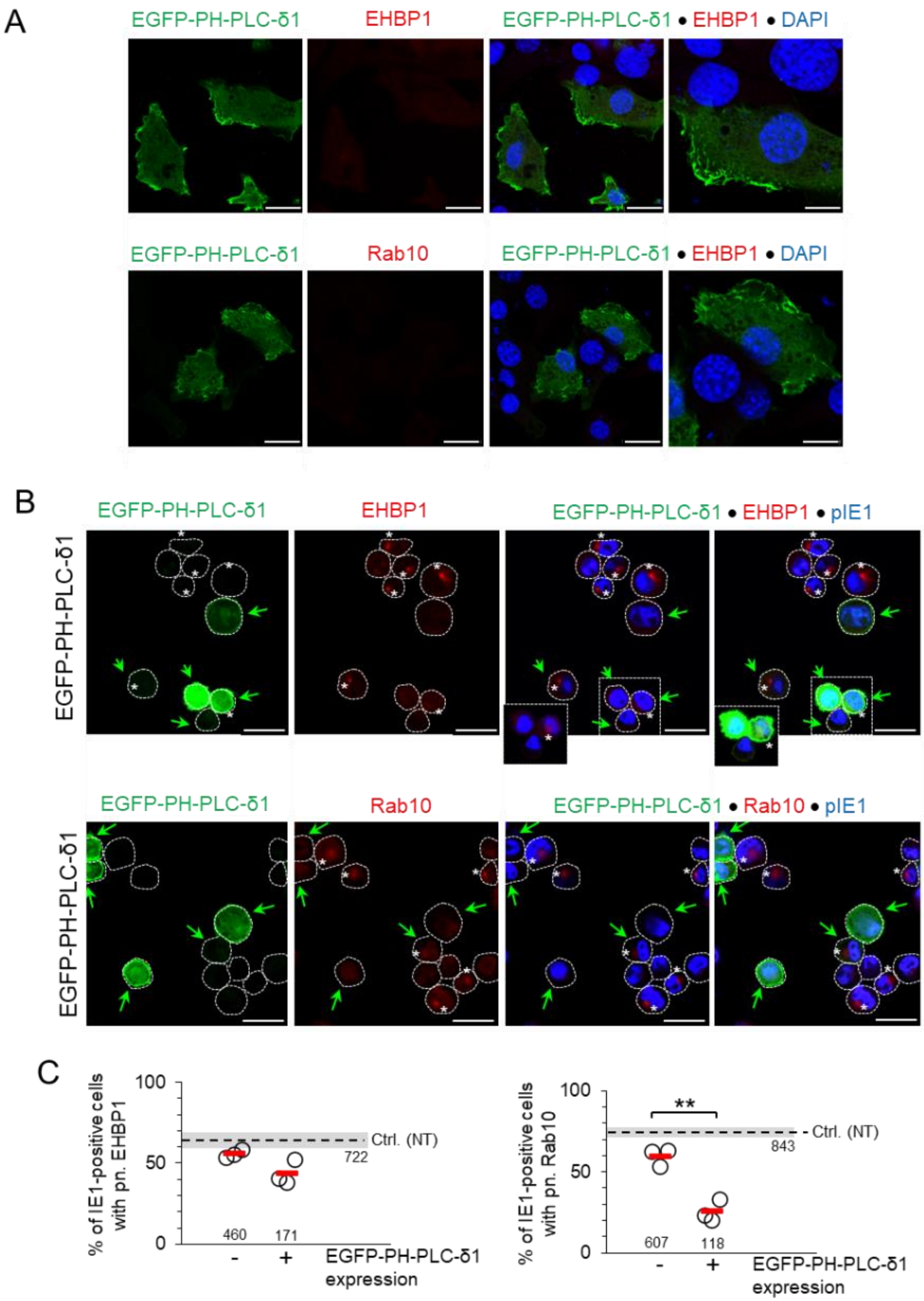

Figure S12. Saturation of PI(4,5)P2 domains by (over)expression of EGFP-PH-PLC-δ1 inhibits membrane recruitment of EHBP1 and expansion of Rab10-PD in Balb 3T3 cells. (**A**) Balb 3T3 cells were transfected with the MSCV expressing EGFP-PH-PLC-δ1 construct and analyzed for expression

of the construct (green) and either endogenous EHBP1 or Rab10 (red) by confocal imaging 24 hours after transfection. Cell nuclei were stained with DAPI (blue). (**B**) MSCV-EGFP-PH-PLC-δ1-transfected cells (24 hours post-transfection) were infected with Δm138-MCMV (MOI 10) for 16 hours and analyzed for expression of the EGFP-PH-PLC-δ1 construct (green), endogenous EHBP1 or Rab 10 (red), and virus-encoded pIE1 (blue). Shown are the focal plane images of green, red, red with blue and all three colors in MCMV-infected cells. Green arrows indicate cells expressing EGFP-PH-PLC-δ1 and white asterisks (*) indicate cells developing perinuclear accumulation of EHBP1 or Rab10. (**C**) Percentage of MCMV-infected (IE1-positive) MSCV-EGFP-PH-PLC-δ1-treated cells non-expressing (-) or expressing (+) EGFP-PH-PLC-δ1 with perinuclear (pn.) accumulation of EHBP1 and Rab10 at 16 hpi. Shown are individual experiments (circles) and mean values (red bars). Ctrl. (NT), control level in non-treated cells (mean ± SD). The numbers indicate the total number of cells analyzed. Statistical significance was determined using the Student t-test (**$p<0.01$).

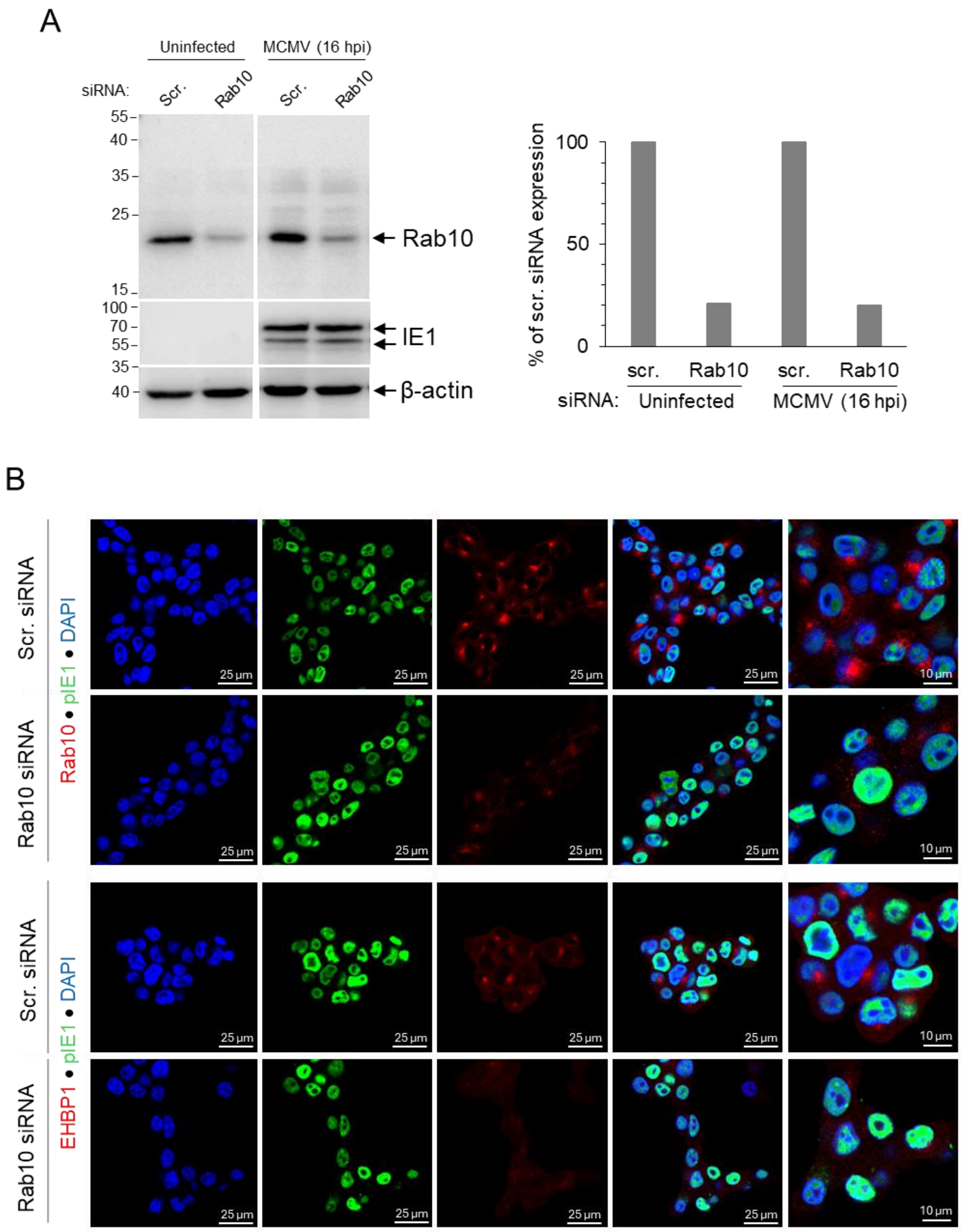

A
Uninfected
MCMV (16 hpi)
siRNA:
Scr.
Rab10
Scr.
Rab10
55
40
35
25
15
100
70
55
35
40
Rab10
IE1
β-actin
% of scr. siRNA expression
100
50
0
scr.
Rab10
scr.
Rab10
siRNA:
Uninfected
MCMV (16 hpi)
B
Scr. siRNA
Rab10 siRNA
Rab10 • pIE1 • DAPI
Scr. siRNA
Rab10 siRNA
EHBP1 • pIE1 • DAPI
25 µm
10 µm

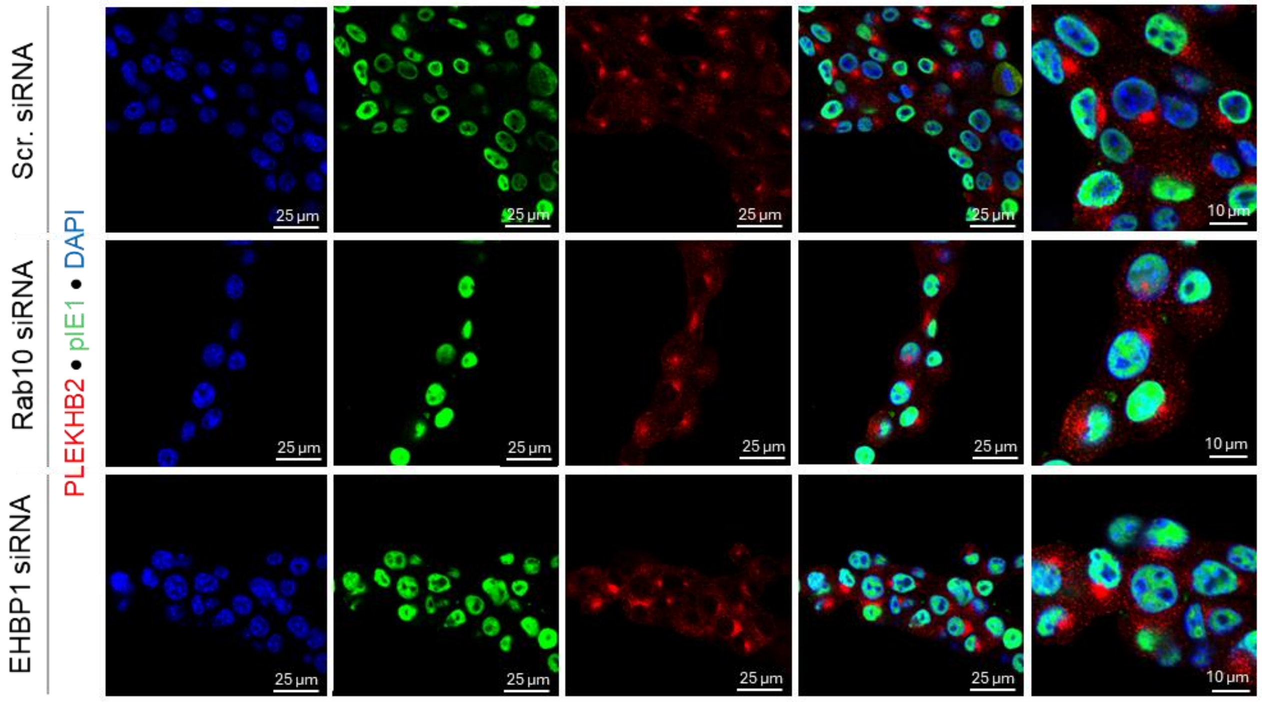

Figure S13. Depletion of Rab10 and EHBP1 does not prevent the formation of pre-AC and tubular intermediates downstream in the EE-to-ERC pathway (*related to Figure 7*). (**A**) Depletion of Rab10 with siRNA. Cells were transfected with siRNA against Rab10 or Scr siRNA (30 nM). After 48 hours, cells were infected with Δm138-MCMV (MOI of 10) for 16 hrs or left uninfected. Expression of Rab 10 was analyzed by Western blot on the same membrane together with pIE1, as a control for infection, and β-actin, as a loading control. Signals on the Western blot were quantified using the ImageJ program, normalized to actin and to Rab10 levels in the scr. siRNA control. (**B**) Rab10, EHBP1 and Scr siRNA-transfected cells were infected with Δm138 MCMV (MOI of 10) and 16 hours after infection were fixed, permeabilized and stained with either Abs against Rab10, EHBP1 or PLEKHB2 (red fluorescence) in combination with Abs against pIE1 for infection control (green) and DAPI (blue) to stain the nuclei.

3.2 Supplementary video

Video S1. The expansion of Rab10-PD in the pre-AC of CMV-infected cells. Expression of EGFP-Rab10 in the NIH 3T3 EGFP-Rab10 cell line was induced by doxycycline (2 µg/ml), and after 24 hours, cells were infected with Δm138-MCMV (MOI of 10). Cells were imaged from 6 hpi with DHTM/fluorescence. Label-free holotomographic time-lapse images (every 2.5 minutes) are shown on the right, fluorescence time-lapse images (every 5 minutes) are shown in the centre, and fluorescence and refractive index overlay are shown on the left. The video refers to Figure 1C.

Video S2. The expansion of Rab10-PD in the pre-AC of CMV-infected cells. Expression of EGFP-Rab10 in the NIH 3T3 EGFP-Rab10 cell line was induced by doxycycline (2 µg/ml), and after 24 hours, cells were infected with Δm138-MCMV (MOI of 10). Cells were imaged from 6 hpi with DHTM/fluorescence. Label-free holotomographic time-lapse images (every 2.5 minutes) are shown on

the right, fluorescence time-lapse images (every 5 minutes) are shown in the centre, and fluorescence and refractive index overlay are shown on the left. The video refers to Figure S4.

Video S3. Recruitment of EGFP-Rab10Q68L to Rab10-PD in the pre-AC. Expression of GTP-locked Rab10 in the NIH 3T3 EGFP-Rab10Q68L cell line was induced by doxycycline (2 μg/ml), and after 24 hours, cells were infected with Δm138-MCMV (MOI of 10). Cells were imaged from 6 hpi with DHTM/fluorescence. Label-free holotomographic time-lapse images (every 2.5 minutes) are shown on the right, fluorescence time-lapse images (every 5 minutes) in the centre and fluorescence and refractive index overlay on the left.

Video S4. The expansion of Rab10-PD in the pre-AC of cells infected with wild-type MVMV. Expression of EGFP-Rab10wt in the NIH 3T3 EGFP-Rab10$_{wt}$ cell line was induced by doxycycline (2 μg/ml), and after 24 hours, cells were infected with wild-type MCMV (MOI of 10). Cells were imaged from 6 hpi with DHTM/fluorescence. Label-free holotomographic time-lapse images (every 2.5 minutes) are shown on the right, fluorescence time-lapse images (every 5 minutes) in the centre and fluorescence and refractive index overlay on the left.

3.3 Supplementary Tables

Table S1. Quantification of Rab10 positive cells in NIH 3T3 cells transfected with scrambled, EHBP1 or Rabin8 siRNA (*related to Figure 5*).

% of pIE$^+$ cells		siRNA		
		**Scrambled**	**EHBP1**	**Rabin8**
Rab10$^+$	Mean ± SD	65.1 ± 2.0	18.8 ± 8.4	60.7 ± 4.2
	P	-	P** = 0.01	P = 0.19

NIH 3T3 cells were non-transfected or transfected with scrambled, EHBP1 or Rabin8 siRNA. 16 hpi after immunofluorescence labeling, the percentage of Rab10$^+$ cells were calculated from three independent experiments, and the results are shown as mean ± SD. Significance of differences from control siRNA-treated samples was determined using Student's t-test (**P<0.01).

Table S2. Quantification of EHBP1 and Rab10 positive cells in non-transfected NIH 3T3 cells and NIH 3T3 cells transfected with MSCV containing EGFP-PH-PLC-δ1 construct (*related to Figure 6*).

% of pIE^{+} cells		EGFP-PH-PLC-δ1 untreated cells	EGFP-PH-PLC-δ1 treated cells	
		**Non-transfected**	**EGFP-PH-PLC-δ1^{-}**	**EGFP-PH-PLC-δ1^{+}**
EHBP1^{+}	Mean ± SD	62.4 ± 6.1	50.4 ± 7.7	27.1 ± 8.3
	P	N.D.	P* = 0.0236	
Rab10^{+}	Mean ± SD	61.3 ± 9.3	48.3 ± 2.7	22.7 ± 2.7
	P	N.D.	P*** = 0.000317	

NIH 3T3 cells were not transfected or transfected for 24 hours with MSCV containing EGFP-PH-PLC-δ1 constructs and infected with Δ138 MCMV (MOI 10). After immunofluorescence labeling at 16 hpi, the percentage of EGFP^{+} and Rab10^{+} cells in transfected and non-transfected pIE1^{+} (MCMV-infected) cells from three independent experiments was calculated, and the results are presented as mean ± SD. Significance of differences was determined using Student's t-test (***$P < 0.001$, *$P < 0.05$)

____

Table S3. Quantification of EHBP1 and Rab10 positive cells in non-transfected and MSCV EGFP-PH-PLC-δ1-transfected Balb 3T3 cells (*related to Figure S12*).

% of IE^{+} cells		EGFP-PH-PLC-δ1 untreated cells	EGFP-PH-PLC-δ1 treated cells	
		**Non-transfected**	**EGFP-PH-PLC-δ1^{-}**	**Non-transfected**
EHBP1^{+}	Mean ± SD	60.8 ± 7.5	55.9 ± 2.5	43.7 ± 7.4
	P	N.D.	P = 0.11	
Rab10^{+}	Mean ± SD	72.0 ± 3.3	59.8 ± 5.4	25.8 ± 6.7
	P	N.D.	P** = 0.0024	

Non-transfected and MSCV EGFP-PH-PLC-δ1 transfected (24 hours post-transfection) Balb 3T3 cells were infected with Δ138 MCMV (10 MOI). After immunofluorescence labeling at 16 hpi, the percentage of EGFP^{+} and Rab10^{+} cells in transfected and non-transfected IE1^{+} (MCMV-infected) cells from three independent experiments were calculated, and the results are reported as mean ± SD. Significance of differences from untreated samples with the same kinetics was determined using Student’s t-test (**P < 0.01).
